# Fire-mediated germination syndromes in *Leucadendron* (Proteaceae) and their functional correlates

**DOI:** 10.1007/s00442-021-04947-2

**Published:** 2021-06-23

**Authors:** Rosemary J. Newton, Berin D. E. Mackenzie, Byron B. Lamont, Pablo Gomez-Barreiro, Richard M. Cowling, Tianhua He

**Affiliations:** 1 Conservation Science, Royal Botanic Gardens Kew, Wakehurst, Ardingly, West Sussex, RH17 6TN UK; 2 Science Division, NSW Department of Planning, Industry and Environment, Locked Bag 5022, Parramatta, NSW 2124 Australia; 3 Centre for Ecosystem Science, University of New South Wales, Kensington, NSW 2052 Australia; 4 Ecology Section, School of Molecular and Life Sciences, Curtin University, PO Box U1987, Perth, WA 6845 Australia; 5 Department of Botany, Nelson Mandela Metropolitan University, PO Box 7700, Port Elizabeth, 6000 South Africa

**Keywords:** Bayesian inference, Heat, Serotiny, Smoke, Soil seed bank

## Abstract

A mechanistic understanding of fire-driven seedling recruitment is essential for effective conservation management of fire-prone vegetation, such as South African fynbos, especially with rare and threatened taxa. The genus *Leucadendron* (Proteaceae) is an ideal candidate for comparative germination studies, comprising 85 species with a mixture of contrasting life-history traits (killed by fire vs able to resprout; serotinous vs geosporous) and seed morphologies (nutlets vs winged achenes). Individual and combined effects of heat and smoke on seed germination of 40 species were quantified in the laboratory, and Bayesian inference applied to distinguish biologically meaningful treatment effects from non-zero, but biologically trivial, effects. Three germination syndromes were identified based on whether germination was dependent on, enhanced by, or independent of direct fire cues (heat and smoke). Seed storage location was the most reliable predictor of germination syndromes, with soil-stored seeds c. 80% more likely to respond to direct fire cues (primarily smoke) than canopy-stored seeds. Notable exceptions were *L. linifolium*, with an absolute requirement for smoke to germinate (the third serotinous species so reported), and two other serotinous species with smoke-enhanced germination. Nutlet-bearing species, whether serotinous or geosporous, were c. 70% more likely to respond to fire cues than winged seeds, but there was no evidence for an effect of phylogeny or persistence strategy on germination. This comprehensive account of seed germination characteristics and identification of germination syndromes and their predictors, supports propagation, conservation and restoration initiatives in this iconic fynbos genus and other fire-prone shrubs with canopy or soil-stored seeds.

**Supplementary Information:**

The online version contains supplementary material available at 10.1007/s00442-021-04947-2.

## Introduction

Sclerophyll shrublands, called fynbos, are the predominant vegetation type in the biodiverse Cape Floristic Region of South Africa (Bergh et al. 2014). As is the case for vegetation types in other Mediterranean-type regions of the world, fynbos is highly fire-prone (Rundel et al. 2016). Wildfires usually occur during the dry summer-autumn period and have a mean return interval of 10–20 years (Kraaij and van Wilgen 2014). In addition to periodic disturbance by fire, fynbos is characterised by nutrient-poor soils and a Mediterranean-type climate with wet winters and dry summers (Rundel et al. 2016). Fynbos species possess a variety of life-history traits that enable fire survival and consequent persistence in the landscape (Kraaij and van Wilgen 2014), and the origin of fire-adapted traits here can be traced back to at least 90 million years (Lamont and He 2012; He et al. 2016). Most plant species in fynbos, as in other fire-prone vegetation, maintain some form of persistent seed bank that is stored in either the canopy (serotiny) or the soil (geospory) and is stimulated to be released and/or germinate in response to fire (Enright et al. 2007; Keeley et al. 2011). These adaptations take advantage of the optimal conditions for seedling recruitment in the postfire environment, and the origin of many can be traced back to the ‘fiery’ Cretaceous (Lamont et al. 2019).

Research so far concerning fire-cued germination has concentrated on the role of direct fire cues such as heat pulses, charred wood, ash and smoke in inducing germination of geosporous species (Newton et al. 2006; Moreira et al. 2010; Downes et al. 2015; Hall et al. 2017). Less attention has been given to the indirect effects of fire, including changes in chemical and nutrient availability in soil following a fire that promote germination (Pons 1989; Preston and Baldwin 1999), soil water changes (Pérez-Fernández et al. 2000), or widened diurnal surface temperatures due to the reduced cover (Brits 1987; Santana et al. 2013), for example. Few studies consider both independent and combined effects of multiple fire-related and environmental cues that alleviate dormancy and/or promote germination (Thomas et al. 2007, 2010; Mackenzie et al. 2016). Research on fire-stimulated germination of serotinous species has focussed largely on the release of seeds from fruits and cones by fire (Causley et al. 2016; Lamont et al. 2020). The possible effects of heat and smoke on germination of serotinous species have rarely been considered (Midgley and Viviers 1990; Dixon et al. 1995; Hanley and Lamont 2000; Ne’eman et al. 2009) even though serotinous seeds are exposed to a heat pulse during the passage of fire (Bradstock et al. 1994) and, once released, to water-soluble smoke chemicals adsorbed by soil particles and released following rainfall (Preston and Baldwin 1999).

The genus *Leucadendron* (Proteaceae) is an important component of fynbos with 85 species and numerous subspecies recognised (Williams 1972; Rebelo 2001). Alarmingly, 51 of these taxa are threatened with extinction with a further 13 taxa considered near threatened (SANBI 2017). The genus consists of dioecious evergreen shrubs that may be locally dominant or emergent in some communities (Cowling and Holmes 1992). *Leucadendron* arose about 30 million years ago (Sauquet et al. 2009) at a time when fire-adapted traits were beginning to diversify during the Cenozoic (Lamont and He 2017). Consistent with other Cape Proteaceae, many *Leucadendron* species (47%) release their seeds at maturity while the remainder retain their seeds in cones. The degree of serotiny is relatively weak (typically 1–4 years’ retention; Williams 1972; Bond 1985; Midgley and Enright 2000) compared with other fire-prone floras (e.g. southwestern Australia, where seed retention among Proteaceae can exceed 10 years; Enright et al. 1998). Hence, even among serotinous *Leucadendron* species, there may be substantial seed release in the absence of fire, and this is increasingly likely in older individuals (Mustart et al. 1994). Serotinous fruits are either flattened, winged achenes that are dispersed by wind, or rounded nutlets that drop to the ground following their release. Following their release after fire, serotinous seeds are blown or washed into litter microsites and become covered by soil, ash and charred plant remains to a mean depth of 5–15 mm (Bond 1985; Lamont et al. 1993, BBL personal observations). Non-serotinous fruits are always nutlets and these are usually collected from the soil surface and stored underground by ants, rodents and, possibly, dung beetles (Bond and Slingsby 1983; Midgley et al. 2002, 2015). Phylogenetic trait-assignment studies indicate that serotiny with winged achenes is the ancestral condition, followed by serotinous nutlets, with soil-stored nutlets the most derived condition (Lamont and He 2012). *Leucadendron* fruits are indehiscent, containing a single seed fused to the pericarp (Williams 1972), and are, hereafter, referred to as seeds.

Following release, seeds of most serotinous species are typically short-lived (< 2 years) and germinate readily in the first wet season (Williams 1972; Holmes and Newton 2004; Lamont et al. 2020). Serotinous cones are exposed briefly to the high temperatures of a fire that kills the plant or branch, with subsequent desiccation resulting in cone opening and seed release (Rebelo 2001). Simulation of this type of heat (650 °C for 90 s, internal cone temperature reaching 47 °C) enhanced seed germination in three *Leucadendron* species (72% increase in *L. conicum*, 20% increase in *L. eucalyptifolium* and *L. uliginosum*) but decreased germination in one species (60% decrease in *L. salignum*) compared with the non-heated controls. However, as the only resprouter here, *L. salignum* is not completely dependent on seeds for regeneration (Midgley and Viviers 1990). Aqueous smoke has been shown to enhance germination by 19–41% (compared with the non-smoked constant temperature controls) in four serotinous species (*L. coniferum*, *L. rubrum*, *L. salignum* − Brown et al. 2003, and *L. conicum* − Brown and Botha 2004). These reports of enhanced germination are surprising given that seeds of serotinous species (where seed retention in the crown is ≥ 1 year) are typically non-dormant after release (Baskin and Baskin 2014).

Early studies on germination of soil-stored *Leucadendron* seeds adopted an ad hoc physiological approach, examining water-soluble inhibitors (Brown and van Staden 1971; van Staden and Brown 1972), the effect of scarification, leaching, light, stratification, oxygen and applied hormones (Brown and van Staden 1973a, b; van Staden and Brown 1973; Brown and Dix 1985), and the effect of H_2_O_2_ (Brits 1986a). Subsequent studies switched to a more ecological approach, focussing on the direct effects of fire on germination. Nutlets of geosporous *Leucadendron* species possess a thick woody pericarp that protects the seed from predation (Rusch et al. 2013), microbial decay (Brown and van Staden 1973a, b; Lamont and Milberg 1997) and from the heat of a fire (Williams 1972) that can exceed 700 °C at the soil surface and 80 °C at a depth of 40 mm (Newton et al. 2006). Heat has received little attention as a germination cue in soil-stored *Leucadendron*, although Williams (1972) noted its positive effects on *L. sessile* (increase of 18–31%) and *L. tinctum* (increase of 20–55%). Aqueous smoke has been shown to enhance germination by 26–62% in two soil-stored species (*L. arctuatum* and *L. tinctum*; Brown and Botha 2004). Unlike species in the Fabaceae that exclude water entry via an impermeable seed coat, *Leucadendron* seeds do not appear to possess physical dormancy as usually understood (Baskin and Baskin 2014). However, positive effects of scarification and seed-coat removal, often used to break physical dormancy, have been demonstrated in *L. tinctum* (Brown and Dix 1985). This is consistent with the ‘oxygen-impermeable physical dormancy’ described for the closely related genus *Leucospermum* (Brits and Manning 2019) that is more widely accepted as physiological dormancy (as defined by Baskin and Baskin 2014), given that the seed coat is thick but water-permeable (thereby precluding physical dormancy). For the vast majority of nutlet-bearing *Leucadendron* species, however, germination requirements remain poorly understood even though such information is essential for their effective propagation and conservation management, especially of rare and threatened taxa.

Strong diurnal temperature fluctuations that occur in soil over summer or following vegetation removal by fire have received some attention as germination cues (Brits 1986b, 1987; Auld and Bradstock 1996; Moreira et al 2010; Santana et al. 2013). Brits (1986b) has studied the likely temperature fluctuations in pre- and post-burn surface soil in detail. This work showed for the hottest month of the year, at a soil depth of 5 mm, that the warmest 12 h period of the day averaged 28 °C and coolest 12 h averaged 22 °C under heavy shade, 41 °C and 22 °C under light shade and 47 °C and 28 °C for mineral soil exposed after fire. There is evidence among some species of a minor increase in the level of germination when subjected to such conditions (Moreira et al. 2010) or even a decrease among others (Luna 2020). Increased alternating postfire temperatures can break physical dormancy to a limited extent (Ooi et al. 2012) or cause endotesta tearing in *Leucospermum*, facilitating increased oxygen supply to the embryo, thereby promoting germination (Brits et al. 1993). These dormancy-breaking effects need to be distinguished from incubation conditions for germination where low (≤ 11 °C; Yan et al. 2001) or cooler fluctuating temperatures, such as 20/10 °C (Brown and Botha 2004), promote germination. Such temperatures ensure germination occurs in late autumn/winter following substantial rain, thereby avoiding responding to unseasonal rainfall during the usual summer drought (Bond 1984; Mustart and Cowling 1993).

*Leucadendron* is the only genus in the world known to possess (a) species with either plant-stored or soil-stored seeds, and (b) plant-stored seeds that are either flat and winged or rounded nutlets, with equivalent soil-stored nutlets also well-represented in this genus (Rebelo 2001; Thuiller et al. 2004; Tonnabel et al. 2014). This provides a unique opportunity for comparative germination studies unfettered by the potential confounding effects of different phylogenetic and biogeographic histories on plant traits. Thus, this background shows that we have (i) unexpected increases in germination of some serotinous *Leucadendron* species following heat or smoke treatments, (ii) an opportunity to test germination requirements of geosporous species that, in general, are poorly known and (iii) great variation in seed morphology and seed bank types within a single genus.

Within this context, we investigated the individual and combined effects of heat and smoke on germination of 40 species comprising a mixture of life-history and functional traits, including 22 species for which no previously published germination data were available. We focused on simulating conditions that seeds would be exposed to under field conditions in the postfire environment and asked the following: (1) What are the individual and combined effects of heat and smoke on germination? (2) What types of germination syndromes can be identified? (3) How well do phylogeny and life-history traits such as persistence strategy, seed morphology and seed storage location correlate with these syndromes?

## Materials and methods

### Species and seed source

Seeds of 40 *Leucadendron* species collected from wild plants in the Western and Eastern Cape Provinces of South Africa between 2001 and 2014 were used in this study (Table S1; authorities, subspecies and varieties are provided here). Cones, no older than 4 years, were collected from a minimum of 40 individual plants, except *L. flexuosum*, *L. meridianum*, *L. loranthifolium* and *L. foedum* where 25, 20, 10 and two plants were sampled, respectively, due to small population size. The species were selected to represent all lineages within the genus based on Tonnabel et al. (2014) and as far as possible included a reasonable sample across the genus of contrasting seed bank types (canopy-stored, soil-stored), seed morphologies (nutlets, winged achenes) and persistence strategies (resprouter, non-resprouter) (Table S1). Limited seed collection opportunities (nutlets have to be collected when they are mature but before they are released) coupled with restricted availability of suitable collections already held at the MSB, resulted in under-representation of soil-stored nutlets. Seeds were sent to the Millennium Seed Bank at Wakehurst in West Sussex, England, and dried to equilibrium at 15% RH and 18 °C. Except for the four species collected in 2014 (the year of the experiments), dried seeds, collected between 2001 and 2008, were stored at international gene bank standards of − 20 °C, thus greatly reducing the risk of seed viability loss in these collections prior to their use.

### Imbibition tests

A subset of 13 species (*n* = 10 seeds per species) including canopy- and soil-stored nutlets and winged seeds (Table S1) were tested for coat permeability by weighing seeds before and after immersion in water for 96 h.

### Germination experiments

#### Experimental design

A fully orthogonal design was used to test the individual and combined effects of direct fire cues (heat pulse and smoke) on postfire germination of 37 *Leucadendron* species (Experiment 1, Table S1). For three additional species, only the combined effect of heat pulse and smoke was examined due to limited seed availability (Experiment 2, Table S1). A postfire after-ripening treatment (8 weeks of dry storage at 12 h/12 h alternating temperatures of 40/20 °C with 12 h/12 h light/dark) was applied to all seeds in Experiments 1 and 2. This was intended to simulate postfire summer temperatures beneath the soil surface (Auld and Bradstock 1996), likely to be encountered following burial of serotinous seeds by soil and litter (Lamont et al. 1993) and burial of geosporous seeds by ants (Bond and Slingsby 1983) or rodents (Midgley et al. 2002). Seeds in the control treatment received only the postfire after-ripening treatment. Seeds in the heat-only treatment and the combined heat plus smoke treatment received a heat treatment (described below) prior to the postfire after-ripening treatment. Seeds in the smoke-only treatment and the combined heat plus smoke treatment, received a smoke treatment (described below) after the postfire after-ripening treatment, consistent with the release of smoke chemicals adsorbed by soil particles at the onset of the wet season (Preston and Baldwin 1999). Five replicates of 50 seeds were used in each treatment except for eight species in which five replicates of 25 seeds were used (Table S1). For each species, each replicate was set up independently of the others and at weekly intervals, such that the first replicate was placed in incubators in week 1 and the fifth and last replicate was placed in incubators 5 weeks later.

#### Heat pulse treatment

Replicate batches of seeds requiring the heat pulse treatment were independently, and at weekly intervals over a period of five weeks, heated to 80 °C for 20 min inside a temperature-controlled incubator. This treatment was chosen to simulate the heat pulse from a heathland fire passing over seeds up to a depth of 40 mm (Newton et al. 2006), after considering results in Keeley and Bond (1997) and Hanley and Lamont (2000), and to avoid potentially lethal temperatures (Moreira et al. 2010). The temperature within the incubator was monitored with a thermocouple thermometer to ensure it remained within 2 °C of the set temperature. Following treatment, seeds were equilibrated to ambient temperature in the laboratory before being subjected to the postfire after-ripening treatments described above.

#### Smoke treatment

Regen2000® Smokemaster liquid smoke solution (Batch number 11468, Grayson, Australia) was filtered through a sterile 0.2 μm Nalgene cellulose acetate × 50 filter syringe (Fisher Scientific, Loughborough, UK) to exclude microbes and particulates prior to dilution with distilled water (1:10, as per manufacturer instructions) for smoke treatments. At the end of the postfire after-ripening treatment, replicate batches of seeds were placed in separate individual Coulter® Counter containers and completely immersed in at least 10 mL (or up to a maximum of 20 mL for larger seeds) of dilute smoke solution (for seeds receiving smoke) or distilled water (controls). The containers were sealed and incubated at 20 °C for 24 h, after which seeds were removed and blotted dry. The replicates for each species were prepared independently of the others and at weekly intervals, and fresh smoke solution was prepared each week.

#### Germination tests

Following the above pre-treatments, seeds were sown on 1% distilled water agar in 90 mm Petri dishes (except for large-seeded *L. argenteum*, *L. loranthifolium* and *L. tinctum* that were sown in square polyethylene containers measuring 100 × 100 × 20 mm). These were then placed in temperature-controlled incubators at 20/10 °C (12 h light/12 h dark) to simulate near-surface soil autumn–winter temperatures in post-fire fynbos known to promote germination (Brits 1986b, 1987; Brown and Botha 2004). Germination was assessed at regular intervals (initially daily and then twice weekly or weekly). Seeds were scored as germinated and removed once the radicle was longer than 2 mm. Seeds visibly deteriorated because of fungal contamination were assumed to be non-viable and were removed, while the remaining seeds were placed on fresh agar to prevent fungal spread. Germination tests were terminated once there was no germination in any replicate for at least 4 weeks and any remaining seeds were dissected to assess seed fill and viability. Firm seeds containing an embryo were considered viable while seeds that had deteriorated during the germination test were considered non-viable. Germination was calculated relative to viable seeds sown to ensure poor germination was due to a lack of germination response rather than variable seed viability.

### Data analyses

Germination data were analysed using Bayesian inference given a number of key advantages over classical frequentist inference involving Null Hypothesis Significance Testing (NHST). These include the following: (i) richer and more informative inferences through the provision of complete distributional information for all model parameters and related functions via the posterior distribution; (ii) reliance on the data actually observed instead of *p-*values and confidence intervals based on hypothetical unobserved data; (iii) the ability to *accept*, as well as reject, null values; (iv) the ease with which functions of model parameters (including treatment differences) and their uncertainty can be quantified *exactly*; and (v) the insensitivity of the posterior distribution to intended or post hoc comparisons (Kruschke et al. 2012). Furthermore, Bayesian inference provides a practical solution to the problem of separation (perfect prediction of a binary response variable by one or more predictors, as in 0% or 100% germination in one or more experimental treatments) often encountered in logistic regressions.

The number of seeds germinated (*G*_*i*_) out of the number of viable seeds (*V*_*i*_) in each Petri dish (*i*) was assumed to follow a binomial distribution with probability *π*_*i*_, and was analysed using generalised linear models (GLMs) with a logistic link function, as follows:

$$G_{i} \sim {\text{Binomial}}\left( {\pi_{i} ,V_{i} } \right)$$

$${\text{logit}}\left( {\pi_{i} } \right) = \eta_{i}$$

(i) $$\eta_{i} = \beta_{0} + \beta_{1} \times H_{i} + \beta_{2} \times S_{i} + \beta_{3} \times H_{i} \times S_{i} \,\,\,\left( {{\text{Experiment}}\,1} \right),\,{\text{or}}$$
(ii) $$\eta_{i} = \beta_{0} + \beta_{1} \times HS_{i}\,\, \,\left( {{\text{Experiment }}2} \right)$$

where *η* is the logit proportion of viable seeds that germinated, *H* is the effect of heat, *S* is the effect of smoke, *H* × *S* is the interaction between heat and smoke, *HS* is the effect of the combined heat plus smoke treatment, and *β*_0_–*β*_3_ are regression coefficients with *β*_0_ (the intercept) corresponding to the control treatment in each experiment. The proportion of viable seeds germinated, *π*_*i*_, is given by $e^{{\eta_{i} }} /{ }\left( {1{ } + { }e^{{\eta_{i} }} } \right)$.

Model parameters were estimated using Bayesian Markov Chain Monte Carlo (MCMC) methods with uninformative priors. Analyses were conducted using the free software R (version 3.5.2, R Core Team 2018) and JAGS (Plummer 2013) via the package ‘R2jags’ (Su and Yajima 2015). Five chains, each comprising 80,000 iterations, were used in the MCMC process with a burn-in of 50,000 iterations and a thinning rate of 10, giving a combined total of 15,000 samples for each posterior distribution. Models were considered to have converged when traceplots were well mixed and the Gelman–Rubin statistics were below 1.1. Estimated sample sizes (ESS) > 10,000 for all regression parameters and derivative functions ensured chain stability and accuracy. Model adequacy was assessed using graphical posterior predictive checks and Bayesian *p*-values (Gelman et al. 1996).

Poor chain convergence and/or strong autocorrelation were addressed by increasing the number of iterations, the thinning rate, and, in some cases, using weakly informative priors (Table S2). Complete separation was addressed using weakly informative Student’s *t* priors (Ghosh et al. 2018; Table S2). Overdispersion (greater variability in the data than is consistent with a binomial model) was addressed using generalised linear mixed models (GLMMs) and the addition of an observation-level random effect for Petri dish, *ε*_*i*_ ~ *N*(0,σ^2^_*i*_), to the GLMs described above. Further details regarding the final model parameters are provided in Tables S3 and S4.

All model parameters and related functions, including germination probabilities, treatment effects (absolute differences in % germination between treatments) and interaction effects (the absolute effect of the interaction term on % germination in Experiment 1) were estimated from the posterior distribution. Modal values were taken as the best estimates and uncertainty was described using 95% Highest-Density Intervals (HDI, the 95% most credible/highest probability density parameter values). Our primary interest was in the magnitude of the differences in % germination between the fire-related treatments and the control. When the HDI of a parameter excludes a target value, that value can be rejected as credible (analogous but not equivalent to a ‘statistically significant difference’ in frequentist NHST). However, this does not imply any *substantive* or *biological* significance (e.g. Lecoutre et al. 2001; Schwab et al. 2011; Wasserstein et al. 2019). We treated an absolute difference in germination probability between treatments of 10% as the minimum effect size of biological interest. Similarly, when assessing the relative contributions of heat and smoke to the maximum germination response, we interpreted control germination ≥ 50% as evidence against an obligate requirement for direct fire cues. Adapting the HDI + ROPE (Region of Practical Equivalence) decision rule (Kruschke 2018), treatment effects with 95% HDIs completely contained within the interval [-10%–10%] (the ROPE) were classified as follows: ‘biologically trivial’ (negligibly different from/practically equivalent to zero); those falling completely outside the ROPE as ‘biologically non-trivial’ (non-negligibly different from zero); and those overlapping the ROPE as ‘uncertain’. Control germination was interpreted in a similar manner using a ROPE of [0%, 50%].

Contingency tables directed at summarising the relationships between fire-stimulated germination and life-history and seed traits were analysed using Bayesian proportional tests via the R package ‘BayesianFirstAid’ (Bååth 2014). To confirm phylogenetic independence of the species sampled, phylogenetic signal among biologically significant germination response to smoke or heat was quantified using Bloomberg’s K and significance tests, based on the *Leucadendron* molecular phylogeny of Tonnabel et al. (2014). Species that were tested for germination response, but not included in the *Leucadendron* phylogeny, were surrogated by ecologically similar species (i.e., *L. nobile* was surrogated by *L. osbornei, L. coniferum* by *L. meridianum* and *L. tinctum* by *L. barkerae*). Analysis of phylogenetic signal was conducted using the R package ‘phytools’ (Revell 2012).

Data of the form X% (Y%, Z%) reported hereafter in the main text and tables are modal values plus 95% HDIs.

## Results

**Fig. 1:**
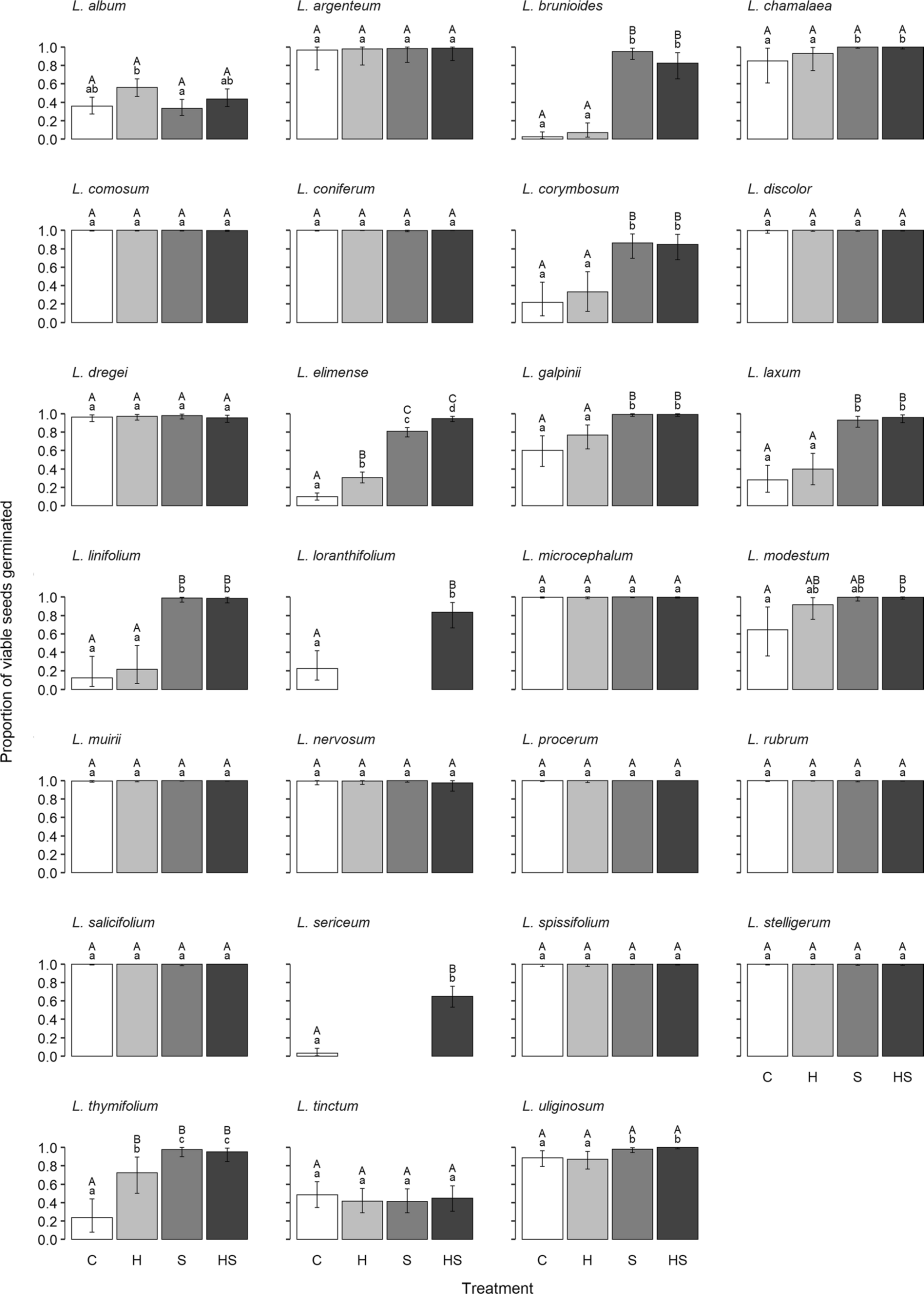
Germination of 27 species of *Leucadendron* (Proteaceae) in response to fire-related germination cues. Treatments are as follows: control (C), heat (H), smoke (S) and heat plus smoke (HS). Data are modal germination ± Bayesian 95% Highest-Density Intervals (quantitative data are provided in Table S5). Different lowercase letters above bars indicate credible non-zero differences between means. Different uppercase letters indicate ‘biologically non-trivial’ differences. Identical uppercase letters indicate either: (i) ‘biologically trivial’ differences, or (ii) insufficient evidence to distinguish biologically trivial from non-trivial effects (see Materials and Methods; Table S7). Individual heat and smoke treatments were not applied to *L. loranthifolium* or *L. sericeum*. The 13 species that germinated to 100% across all experiment treatments have been omitted

Thirteen serotinous species with winged achenes (*L. eucalyptifolium*, *L. flexuosum*, *L. foedum*, *L. gandogeri*, *L. lanigerum*, *L. laureolum*, *L. meridianum*, *L. nobile*, *L. rourkei*, *L. salignum*, *L. strobilinum*, *L. teretifolium* and *L. xanthoconus*) germinated to 100% in every treatment and were, therefore, not subjected to statistical analysis. Of the remaining 27 species, modal germination in the control treatment (summer postfire dry after-ripening treatment of 40/20 °C followed by moist incubation at autumn–winter postfire temperatures of 20/10 °C) exceeded 50% for 17 species but was less than 20% for four species (*L. brunioides*, *L elimense*, *L. linifolium* and *L. sericeum*). Modal germination of all species reached 80–100% in at least one treatment except for *L. album*, *L. sericeum* and *L. tinctum* (Fig. 1, Table S5). Average seed mass for 13 species (Table S1) increased by > 20% (based on air-dried seed mass) for all species tested, indicating that seeds of all of these species are permeable to water, based on criteria in Baskin and Baskin (2003).

### Germination syndromes

**Fig. 2:**
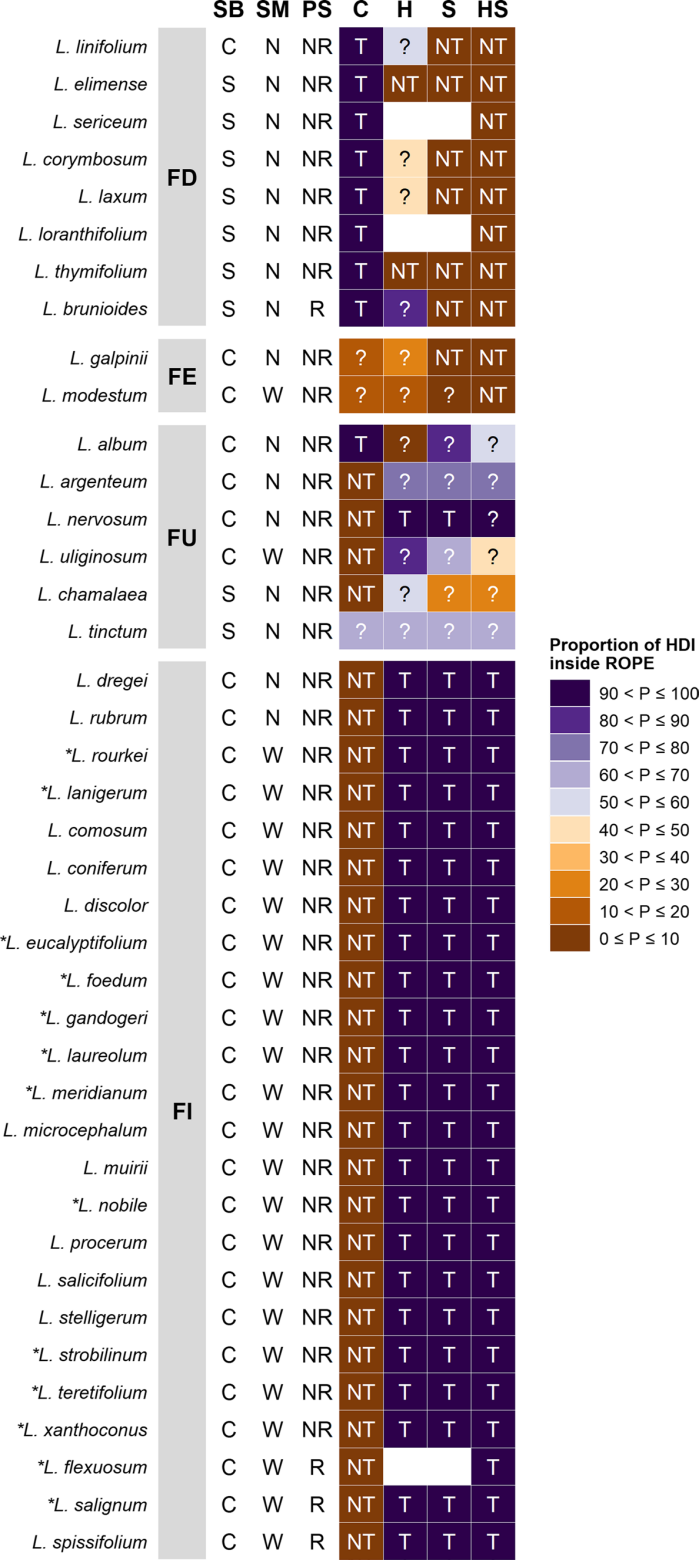
Generalised effects of fire-related cues on germination of 40 *Leucadendron* species arranged by germination, seed trait and regeneration syndromes. Germination syndromes (GS): FD (fire cue-dependent) one or more direct fire (smoke/heat) cues are required for ≥ 50% germination; FE (fire cue-enhanced) one or more direct fire cues have a non-trivial (≥ 10%) promotive effect but germination is (≥ 85%) likely to be ≥ 50% in their absence; FI (fire cue-independent) direct fire cues have trivial (< 10%) effects on germination and germination is ≥ 50% in their absence; and FU (fire cue effects uncertain) insufficient evidence to distinguish trivial from non-trivial fire effects; however (with the exception of *L. album* and *L. tinctum* that had low overall germination), direct fire cues are not required for substantial (≥ 50%) germination. Seed trait syndromes: *SB* seed bank type (*C* canopy-stored, *S* soil-stored); *SM* seed morphology (*N* nutlets, *W* winged achenes). *PS* persistence strategy (*NR* non-resprouter, *R* resprouter). Germination cues: *C* control; *H* heat; *S* smoke; *HS* heat + smoke; *NT* biologically non-trivial response; T –biologically trivial response; ? Uncertain response; blank–treatment not applied. Shading indicates the proportion of the 95% HDI inside the ROPEs for control germination and treatment effects, respectively, except for 13 species (*) where the probabilities of trivial control germination and trivial treatment effects are inferred to be 0 and 1, respectively, on the basis of 100% germination in all treatments. Refer to Materials and Methods for details, and to Table S7 for quantitative data on effect sizes. Life-history and seed trait data obtained from Williams (1972), Rebelo (2001) and Tonnabel et al. (2018)

Several broad germination syndromes were evident in the data (Fig. 2, Table S5, S6): (i) taxa with fire cue-*dependent* germination (*n* = 8), where a heat pulse and/or smoke was required for ≥ 50% germination (mean control germination 2–28%); (ii) taxa with fire cue-*enhanced* germination (*n* = 2), where smoke had a non-trivial promotive effect but germination was 85–88% likely to be ≥ 50% (mean 60–65%) in its absence (the alternative, fire cue-*dependent* germination, was only 12–15% likely); (iii) taxa with fire cue-*independent* germination (*n* = 24), where both heat pulse and smoke had trivial effects and mean control germination was 96–100%; and (iv) taxa with *uncertain* germination responses to fire (*n* = 6), where there was insufficient evidence to distinguish biologically trivial from non-trivial promotive effects of heat and/or smoke; however, germination was not fire cue-*dependent* with a mean control germination of 85–99% (apart from *L. album* and *L. tinctum* that germinated poorly overall). The fire-dependent group is almost entirely geosporous with a single serotinous member (*L. linifolium*). Both the fire-enhanced and fire-independent groups comprise serotinous taxa only, while the uncertain group is a mixture of seed bank types.

### Heat pulse responses

Heat had a non-trivial effect on two geosporous species, *L. elimense* and *L. thymifolium*, improving germination by 21% (14%, 28%) and 47% (18%, 73%), respectively (Fig. 2, Tables S6, S7). The effect of heat was trivial on a further 24 species, all of which were serotinous, but was uncertain for the remaining 11 species. Among the latter, heat had a potentially minor effect on *L. album* [20% (6%, 33%), 94.9% of HDI outside ROPE].

### Smoke responses

Smoke had a positive, non-trivial effect on seven species [including two serotinous species, improving germination by 39% (23%, 56%) to 91% (82%, 97%) (Fig. 2, Tables S6, S7)]. The effect of smoke was trivial on a further 24 species but was uncertain on the remainder, although its effect on *L. modestum* was likely non-trivial [34% (9%, 62%), 99.6% of HDI outside ROPE].

### Responses to combined heat plus smoke

The combined heat plus smoke treatment had a positive, non-trivial effect on the same seven species as the smoke-only treatment plus an additional three species, two of which did not receive a smoke-only treatment. Trivial effects were observed for 24 other species with the effect on an additional species, *L. nervosum*, also likely to be trivial [− 1% (− 12%, 4%), 98.4% of HDI inside ROPE]. The effect of the combined heat plus smoke treatment was uncertain for the remaining species.

The difference in germination between the combined heat plus smoke treatment and the smoke-only treatment was trivial for 28 out of 37 species where both treatments were applied (including 13 species with 100% germination in both treatments; Fig. 2, Tables S6, S7) and uncertain in the remaining 9 species. Minor negative interactions between heat and smoke were observed for *L. brunioides* and *L. thymifolium* (Fig. 1, Table S3). Both interactions had an uncertain effect on germination [*L. brunioides*, − 15% (− 34%, − 2%); *L. thymifolium*, − 4% (− 15%, 0%); Table S7].

### Effects of phylogeny, life-history and seed traits

Non-trivial germination responses to heat pulses and smoke were independent of species phylogenetic relationships (*K* = 0.233, *P* = 0.266; *K* = 0.295, *P* = 0.075, respectively). Germination responses to fire cues (including both promotive effects and absolute requirements) were 80–90% more likely in species with soil seed banks than those with canopy seed banks (Table 1). There was little evidence of a difference in the frequency of fire-stimulated germination between resprouters and non-resprouters. Smoke-responsive species were 90% more likely to respond to heat than non-smoke-responsive species. Fire cues were 72% more likely to promote germination in nutlets than winged seeds. The prevalence of fire effects in nutlet-bearing species was 42% higher in those with soil seed banks versus canopy seed banks, and fire effects in serotinous species were 45% more common in those with nutlets than those with winged seeds (Table 1).

**Table 1:** Summary of germination responses to fire cues in relation to seed bank and seed morphology

	Soil	Canopy
*Fire effects vs seed storage location*		
Fire effect	7	3
No fire effect	0	24
Relative frequency of fire effects	0.978 [0.69, > 0.999]	0.106 [0.033, 0.264]
Difference in prevalence of fire effects between groups: 0.810 [0.514, 0.943]		
Probability of greater prevalence of fire effects in species with soil seed banks: > 0.999		

	Soil	Canopy
*Heat effects vs seed storage location*		
Heat effect	2	0
No heat effect	0	24
Relative frequency of heat effects	0.947 [0.356, > 0.999]	0.007 [< 0.001, 0.116]
Difference in prevalence of heat effects between groups: 0.896 [0.316, 0.995]		
Probability of greater prevalence of heat effects in species with soil seed banks: > 0.999		

	Soil	Canopy
*Smoke effects vs seed storage location*		
Smoke effect	5	2
No smoke effect	0	24
Relative frequency of smoke effects	0.971 [0.599, > 0.999]	0.074 [0.012, 0.216]
Difference in prevalence of smoke effects between groups: 0.829 [0.469, 0.962]		
Probability of greater prevalence of smoke effects in species with soil seed banks: > 0.999		

	Non-resprouter	Resprouter
*Fire effects vs persistence strategy*		
Fire effect	9	1
No fire effect	21	3
Relative frequency of fire effects	0.293 [0.158, 0.472]	0.234 [0.027, 0.669]
Difference in prevalence of fire effects between groups: 0.029 [− 0.407, 0.338]		
Probability of greater prevalence of fire effects in non-resprouters: 0.489		

	Smoke effect	No smoke effect
*Heat effects vs smoke effects*		
Heat effect	2	0
No heat effect	0	24
Relative frequency of heat effects	0.947 [0.356, > 0.999]	0.007 [< 0.001, 0.116]
Difference in prevalence of heat effects between groups: 0.896 [0.316, 0.995]		
Probability of greater prevalence of heat effects in species with smoke effects: > 0.999		

	Nutlet	Winged
*Fire effects vs seed morphology (serotinous and geosporous species combined)*		
Fire effect	9	1
No fire effect	2	22
Relative frequency of fire effects	0.813 [0.54, 0.961]	0.049 [0.003, 0.185]
Difference in prevalence of fire effects between groups: 0.721 [0.442, 0.91]		
Probability of greater prevalence of fire effects in species with nutlets: > 0.999		

	Soil	Canopy
*Fire effects vs seed storage location (nutlet-bearing species only)*		
Fire effect	7	2
No fire effect	0	2
Relative frequency of fire effects	0.976 [0.683, > 0.999]	0.52 [0.152, 0.859]
Difference in prevalence of fire effects between groups: 0.417 [− 0.018, 0.794]		
Probability of greater prevalence of fire effects in species with soil seed banks: 0.964		

	Nutlet	Winged
*Fire effects vs seed morphology (serotinous species only)*		
Fire effect	2	1
No fire effect	2	22
Relative frequency of fire effects	0.496 [0.151, 0.859]	0.049 [0.002, 0.187]
Difference in prevalence of fire effects between groups: 0.448 [0.045, 0.787]		
Probability of greater prevalence of fire effects in species with nutlets: 0.990		

Fire effect refers to a heat and/or smoke effect, and these include both fire cue-enhanced and fire cue-dependent germination responses. Group counts refer to non-trivial and trivial effects of heat and/or smoke only (uncertain effects have been excluded; refer to Table S7 and main text for details). Group frequencies and differences are mode plus Bayesian 95% Highest-Density Intervals (the most credible parameter values). Note that non-trivial germination responses to heat and/or smoke were independent of species phylogenetic relationships

## Discussion

The most important predictor of germination syndromes in *Leucadendron* is seed storage location (serotinous vs geosporous) followed by seed morphology (winged achenes vs nutlets). Excluding four serotinous species with uncertain germination responses in relation to fire cues, 96% (22) of the serotinous, wing-seeded species examined exhibited fire-independent germination and 50% (two) of serotinous, nutlet-bearing species displayed fire-enhanced germination (Table 1). This contrasts with 100% (seven) geosporous, nutlet-bearing species with fire-dependent germination (Table 1). In terms of a possible evolutionary sequence, these traits and the associated germination syndromes reflect increased ‘fine-tuning’ to fire. Fire plays an indirect role in recruitment of species with serotinous, winged fruits (the ancestral condition in *Leucadendron*; Lamont and He 2012; Tonnabel et al. 2018) by timing *en masse* seed release to the immediate postfire environment, although their relatively weak serotiny implies progressive seed release in the absence of fire, but does not manifest as recruitment, with these seeds lost to the seed bank [with the exception of *L. argenteum*, which maintains both serotinous aerial seedbanks and extremely long-lived soil-stored seed banks (Heelemann et al. 2013)]. Recruitment of geosporous species, on the other hand, is tightly coupled to fire, with smoke and (to a lesser extent) heat providing essential germination cues.

### Germination of serotinous species

Serotinous species are widely regarded as non-dormant (Lamont and Enright 2000). However, our study provides evidence that direct fire cues promote germination in a few taxa. Most notably, we identified a serotinous species, *L. linifolium*, with an obligate smoke *requirement* for germination (Figs. 1, 2, Table S6). To our knowledge, this is only the third report of such a species in the literature and is the first report for the South African flora. However, this is contradicted by Yan et al. (2001), who obtained 71% germination under a 20/10 °C cycle of 16 ﻿h light/8 h dark, without any additional cues. Even so, the strong smoke response by *L. linifolium* in our collection is consistent with its membership of the nutlet clade I of Tonnabel et al. (2018) that contains five of the nine species in our fire-dependent/enhanced syndromes (Fig. 2) that respond to smoke. The other two serotinous obligate smoke species occur in southwestern Australia: *Callitris* (*Actinostrobus*) *acuminata* (Cupressaceae) requires smoke to germinate (Dixon et al. 1995) and *Conospermum capitatum* (Proteaceae) requires smoke or heat (Zhao and Ladd 2014). The designation of *L. conicum* as a fourth serotinous species with an obligate requirement for a fire cue (heat) seems reasonable on the basis of Midgley and Viviers (1990), where mean germination increased from 18% in the control to 90% with heat treatment although this is contradicted by Bond (1984), who reported 100% seed germination using untreated seeds of the same species.

Facultative smoke responses were displayed by two other serotinous species, *L. galpinii* [39% (23%, 56%) increase relative to control] and *L. modestum* [34% (9%, 62%) increase] (Fig. 1, Table S7). Similar germination enhancements with smoke treatment were reported by Brown et al. (2003) for *L. coniferum*, *L. rubrum* and *L. salignum*, but these were not supported in our study, with near-full germination of all three species by the control. Reports of mild (c. 20%) improvements in mean germination with smoke treatment of other serotinous Proteaceae include *Protea compacta* (Brown 1993), *Aulax cancellata* (Brown et al. 2003) and *L. conicum* (Brown and Botha 2004). In contrast, Yearsley et al. (2018) observed no effect of smoke on germination of eight serotinous species of Western Australian Proteaceae.

There was no evidence of positive or negative effects of heat on germination of any of the 31 serotinous species examined in our study, including three species for which a positive effect (*L. eucalyptifolium* and *L. uliginosum*, Midgley and Viviers 1990; *L. tinctum*, Williams 1972) and one for which a 60% decrease in germination (*L. salignum*, Midgley and Viviers 1990) were reported previously. Promotive effects (Brown and Whelan 1998; Hanley and Lamont 2000) and nil or strong negative effects (Bell and Williams 1998; Ne’eman et al. 2009) of heat have also been reported for several other serotinous taxa. Reduced responses in these studies were attributed to seed death, though a heat pulse can suppress germination without affecting viability in some species (Collette and Ooi 2017; Luna 2020).

The heat treatment applied in our study (80 °C for 20 min) did not reduce germination in any of the species tested so cannot be considered excessive. Heat tolerance is an essential prerequisite for soil-stored seeds, but it is noteworthy that such tolerance was also evident among all species with plant-stored seeds. This should facilitate survival of seeds stored in cones during the passage of fire when raised temperatures can occur despite insulation by the woody scales (e.g. over 60 °C for up to 15 min inside woody follicles of *Hakea dactyloides*; Bradstock et al. 1994) and of seeds of weakly serotinous *Leucadendron* species that may be released and incorporated into the soil prefire. Survival of seeds without affecting germination following similar heat treatments has been noted among serotinous species in a number of sclerophyll genera (Bradstock et al. 1994; Hanley and Lamont 2000; Hall et al. 2017) as well as non-serotinous species in other regions (Williams et al. 2003; Gómez-González et al. 2017).

### Germination of geosporous species

Germination was tightly cued to fire in the majority of geosporous species with smoke providing the principal germination cue. The heat pulse acted as an alternative, though less effective, cue for two species (*L. elimense* and *L. thymifolium*, Fig. 1, Table S7). Heat and smoke can be distributed patchily during a fire (Auld and Bradstock 1996) and the ability to respond to more than one germination cue has been suggested to maximise the capability of seeds to sense the passage of fire (Kenny 2000). This may be especially important for non-resprouters that rely exclusively on postfire seedling recruitment for population persistence.

Imbibition tests showed all seed types were water-permeable, including those that responded to heat (Table S1), and, therefore, physical dormancy is not a feature of this genus (Baskin and Baskin 2014). The mechanism by which heat promotes germination of hard water-impermeable/physically dormant seeds has been well studied, but its mode of action on water-permeable seeds, such as *Leucadendron*, has received less attention. In the closely related genus, *Leucospermum*, dormancy is imposed by the water-permeable testa restricting oxygen diffusion to the embryo (Brits 1986b). The heat pulse from a fire is thought to alleviate dormancy via desiccation scarification of the testa (Brits et al. 1993; Brits and Manning 2019) and this is consistent with reports of a positive correlation between fire severity, via increased diurnal range of temperatures, and postfire seedling densities in *Leucospermum conocarpodendron* (Bond et al. 1990). Treatments that increase oxygen supply to the embryo, including scarification and high oxygen tensions, promote germination of *Leucadendron daphnoides* (Brown and van Staden 1973b). Heat responses in nut-fruited *Leucadendron* species might also cause alleviation of embryo anoxia in the same way but has yet to be examined. Our observation that smoke in the absence of heat was highly effective in promoting germination of the two heat-responsive *Leucadendron* species, but not for *Leucospermum* species (Brown and Botha 2004), indicates that the mechanisms of physiological dormancy between these genera do differ.

Two exceptions to the clear pattern of fire-dependent germination in species with soil-stored seeds were the uncertain effects of heat and smoke on *L. tinctum* and *L. chamalaea* (Fig. 2, Table S6)*.* The relatively low overall germination of *L. tinctum* (Fig. 1) was possibly due to incomplete alleviation of physiological dormancy and/or application of suboptimal germination cues. In contrast to the present study, Brown and Botha (2004) reported smoke-enhanced germination in this species of 25–62% *cf* the controls, although no estimates of uncertainty were provided. They also reported that combined smoke and scarification improved germination by 25% *cf* smoke alone, but that improvement was negligible (6%) with combined heat and smoke *cf* smoke alone. An increase of germination by 20% following heat treatment was observed by Williams (1972). Germination of the controls in each of these trials was 12–35%, suggesting that germination of *L. tinctum* could, in fact, be fire cue-dependent rather than fire cue-enhanced or fire cue-independent. In *L. chamalaea*, germination of the control (subjected to a summer postfire after-ripening treatment and incubated at autumn postfire temperatures) was unexpectedly high for a geosporous species [85% (61%, 99%), Fig. 1]. Mosime (2016) reported substantial (up to 40–50%) increases in germination of *L. chamalaea* with smoke; however, this was relative to 40% germination of 10 °C and 25 °C constant temperature controls, suggesting our use of diurnal alternating temperatures might have obviated the need for smoke as an alternative cue.

Both *L. tinctum* and *L. chamalaea* are geosporous non-resprouters that rely on seedling recruitment for postfire persistence. A period of in situ burial is sometimes required before seeds become responsive to fire cues (Roche et al. 1997; Baker et al. 2005; Newton et al. 2006) and this is worthy of further research. Additionally, ex situ storage might have unmonitored effects on dormancy and germination responses (Baskin et al. 2006); hence, caution is required when interpreting small or uncertain effects of fire cues observed among ex situ stored seeds. Possible explanations for this discrepancy include differences in the level of primary dormancy, seed age and storage effects, and interaction effects between smoke and temperature (Mackenzie et al. 2016). Further research is needed to resolve these disparate results, including examining other species with large nutlets that may remain dormant during cool fire cycles (noted by Bond et al. 1990).

The present study focussed on germination in the postfire environment and the role of cues directly associated with the passage of fire (i.e. heat pulse and smoke). However, germination of some species may be promoted to a greater or lesser extent by the indirect effects of fire, which would aid competition avoidance by restricting germination to the postfire environment or to canopy gaps in unburnt vegetation. Increased temperature fluctuations can impact seeds over summer (where there is potential for dry after-ripening) as well as over autumn and winter (while seeds are imbibed). Establishing the role of increased temperature fluctuations during these different seasons in *Leucadendron* seed germination requires a comparison of germination responses at under-canopy temperatures with those at gap/postfire temperatures. Nevertheless, all 30 serotinous species, except *L. linifolium* (97%), showed potential for substantial germination in canopy gaps in unburnt fynbos (as indicated by germination of the controls) compared with one (11%) geosporous species, *L. chamalaea* (Figs. 1, 2, Table S6). However, the potential for such germination to result in successful seedling recruitment is low (Bond 1984), with *Banksia serrata* a notable exception (Whelan et al. 1998). The few seedlings that do survive are usually starved of resources and remain depauperate (BBL, RMC, personal observations); fitness depends on survival and subsequent fecundity, not just on germination in the absence of disturbance (Lamont et al. 2019).

### Comparing germination responses and detecting treatment effects

It is clear in this discussion that within species, very different germination results have sometimes been obtained in different studies. Identifying the possible cause of disparate results is tricky, as a multitude of factors that affect variability in primary seed dormancy and viability, both within and between populations and over time, such as maternal effects and phenotypic plasticity, are virtually impossible to predict and control. Furthermore, differing study aims result in different control, pre-treatment and germination conditions being chosen: those selected for an ecological study are likely to differ substantially from a horticultural study attempting to maximize seed germination in the shortest possible time. Finally, conditions during storage and germination affect seed vigour and viability and consequently germination outcomes. It is vital that future studies record and report these factors known to affect seed dormancy and germination (both total and rate), and consider possible interactions, to enable better interpretation of seed germination responses in an ecological context.

Finally, we comment on our approach to identifying important treatment effects. Despite widespread criticism of NHST, it continues to be routinely applied in seed science research to infer the existence of substantive effects on the sole basis of ‘statistical significance’ and, conversely (and erroneously), to infer the non-existence of such effects when faced with non-significant results. Even less desirable is the diagnosis of treatment effects based on point estimates alone while ignoring precision. Here, we advocate a more robust and informative approach as follows, in which: (i) both the magnitude *and* uncertainty of treatment effects are explicitly acknowledged and reported; (ii) consideration is given to the minimum effect size of biological consequence; and (iii) the importance of an effect is interpreted on the basis of its magnitude relative to the total germination response.

In this study, we regarded a change in germination of ± 10 percentage points as the minimum meaningful effect. By explicitly acknowledging the uncertainty in our estimates and requiring at least 95% confidence to discern non-trivial from trivial effects, 16 (34%) of the credibly non-zero effects identified in pairwise contrasts (those with 95% HDIs excluding zero; analogous but not equivalent to ‘statistically significant’ in NHST) were too imprecisely estimated to reach a clear decision (Fig. S1, Table S7). Where non-trivial heat and smoke effects were identified, background (control) germination responses were used to evaluate their relative importance and distinguish supplementary cues from the principal drivers of postfire germination. Furthermore, 92 effects (70%) with zero among their credible values were assessed as biologically trivial (the remaining 30% were too imprecisely estimated to classify either way). These results highlight the utility of Bayesian estimation and the importance of differentiating substantive from statistical significance. While the critical values for the minimum non-negligible effect size and the required degree of confidence will vary between objectives and researcher proclivities, the subjective element can never be eliminated from interpreting data. Greater focus on parameter magnitudes and uncertainty will vastly improve confidence in the biological significance of the results obtained in future germination studies.

### Conclusions

This comprehensive study on seed germination requirements of 40 *Leucadendron* species has shown that the most important predictor of germination syndromes in this important fynbos genus is seed storage location (serotinous vs geosporous) followed by seed morphology (winged achenes vs nutlets). Fires at moderate intervals are essential for (i) releasing the seeds of serotinous species onto an optimal seedbed for germination and recruitment but rarely contribute to their germination, and (ii) stimulating high levels of germination of geosporous species via smoke as they are water-permeable while fire-type heat is sometimes beneficial, possibly as a failsafe mechanism. These findings, in conjunction with established knowledge, such as a low (alternating) temperature requirement for germination, can be used to inform horticultural and endangered-species programs on propagation requirements for conservation and restoration purposes. These results have considerable generality as their wide range covers most likely outcomes among sclerophyll shrubs in South Africa and Australia in particular, but the unexpected exceptions documented here highlight the merit in examining the particular germination requirements of all species under consideration in more detail. Finally, this study highlights the importance of the following: (1) considered study design, including meaningful controls, particularly for understanding seed germination in an ecological context; (2) explicitly reporting factors that affect seed dormancy and germination to enable valid study comparisons; and, (3) using appropriate statistical methods for identifying biologically meaningful responses in seed germination studies.

## Supplementary Information

Below is the link to the electronic supplementary material.

Supplementary file1 (DOCX 144 KB)

Supplementary file2 (TIF 53 KB)

## Data Availability

The datasets used and/or analysed during the current study are available from BDEM on reasonable request.
