# Supplementary material for: Fire-mediated germination syndromes in Leucadendron (Proteaceae) and their functional correlates
Source: Oecologia. 2021 Jun 23;196(2):589–604. doi: 10.1007/s00442-021-04947-2 (PMC8241639; doi:10.1007/s00442-021-04947-2)
Supplement: Supplementary file 1 — Supplementary file1 (DOCX 144 KB) [file 442_2021_4947_MOESM1_ESM.docx]

**Electronic Supplementary Material**

**Table S1.** *Leucadendron* species with MSB serial number, seed trait syndromes: SB – seed bank type (C – canopy-stored, S – soil-stored); SM – seed morphology (N – nutlets, W – winged achenes); PS – persistence strategy (NR – non-resprouter, R – resprouter); conservation status (CR – critically endangered, EN – endangered, VU – vulnerable, NT – near threatened, LC – least concern; SANBI 2017), collection date and numbers of seeds used in germination tests. Experiments 1 and 2 tested the effect of direct fire cues on seed germination: Experiment 1 consisted of four treatments (control, heat, smoke, heat + smoke) while Experiment 2 comprised only two treatments (control, heat + smoke). All seeds in Experiments 1 and 2 were exposed to simulated postfire temperatures of 40/20°C for 8 weeks after heat but before smoke treatments were applied. A subset of 13 species (indicated by an asterisk, n = 10 seeds per species) covering canopy- and soil-stored, nutlets and winged seeds (Fig. 2, Table S6) were tested for water permeability over an imbibition period of 96 h. Seed mass increased by > 20% (based on air-dried seed mass) in all species tested, indicating seeds of these species are permeable to water (Baskin and Baskin 2003) and therefore do not possess physical dormancy (Baskin and Baskin 2014).

| MSB  Serial No. | Species | Seed bank type | Seed morphology | Plant persistence strategy | Conservation status | Collection date | Experiment | Reps × seeds per treatment |
| --- | --- | --- | --- | --- | --- | --- | --- | --- |
| 190013 | ** L. album* (Thunb.) Fourc. | C | N | NR | LC | 06-Feb-2003 | 1 | 5×50 |
| 244145 | ** L. argenteum* (L.) R. Br. | C | N | NR | EN | 10-Feb-2004 | 1 | 5×50 |
| 189761 | ** L. brunioides* Meisn. | S | N | R | LC | 04-Jan-2002 | 1 | 5×50 |
| 457752 | *L. chamalaea* (Lam.) I. Williams | S | N | NR | CR | 21-Feb-2008 | 1 | 5×50 |
| 792439 | ** L. comosum* (Thunb.) R. Br. | C | W | NR | LC | 12-Apr-2014 | 1 | 5×50 |
| 189185 | *L. coniferum* (L.) Meisn. | C | W | NR | VU | 01-Mar-2002 | 1 | 5×50 |
| 214588 | *L. corymbosum* P.J. Bergius | S | N | NR | VU | 17-Jan-2004 | 1 | 5×50 |
| 214810 | *L. discolor* E. Phillips & Hutch. | C | W | NR | VU | 25-Jan-2004 | 1 | 5×50 |
| 792440 | *L. dregei* E. Mey. ex Meisn. | C | N | NR | EN | 11-Apr-2014 | 1 | 5×25 |
| 215068 | ** L. elimense* E. Phillips subsp. *elimense* | S | N | NR | EN | 16-Feb-2004 | 1 | 5×50 |
| 189679 | *L. eucalyptifolium* H. Buek ex Meisn. | C | W | NR | LC | 06-Jan-2003 | 1 | 5×50 |
| 484101 | *L. flexuosum* I. Williams | C | W | R | CR | 28-Feb-2008 | 2 | 5×25 |
| 486057 | *L. foedum* I. Williams | C | W | NR | CR | 21-Jul-2008 | 1 | 5×25 |
| 214278 | ** L. galpinii* E. Phillips & Hutch. | C | N | NR | VU | 18-Dec-2003 | 1 | 5×50 |
| 189521 | *L. gandogeri* Schinz ex Gand. | C | W | NR | LC | 10-Dec-2002 | 1 | 5×50 |
| 214991 | *L. lanigerum* H. Buek ex Meisn. var. *laevigatum*  Meisn. | C | W | NR | CR | 12-Feb-2004 | 1 | 5×50 |
| 189152 | *L. laureolum* (Lam.) Fourc. | C | W | NR | LC | 05-Mar-2002 | 1 | 5×50 |
| 215116 | ** L. laxum* I. Williams | S | N | NR | EN | 17-Feb-2004 | 1 | 5×50 |
| 189196 | ** L. linifolium* (Jacq.) R. Br. | C | N | NR | VU | 04-Mar-2002 | 1 | 5×50 |
| 302508 | *L. loranthifolium* (Salisb. ex Knight) I. Williams | S | N | NR | NT | 29-Nov-2005 | 2 | 5×25 |
| 327170 | *L. meridianum* I. Williams | C | W | NR | LC | 20-Mar-2006 | 1 | 5×50 |
| 213950 | *L. microcephalum* (Gand.) Gand. & Schinz | C | W | NR | LC | 18-Oct-2003 | 1 | 5×50 |
| 389697 | *L. modestum* I. Williams | C | W | NR | EN | 25-Jan-2007 | 1 | 5×25 |
| 188672 | *L. muirii* E. Phillips | C | W | NR | LC | 01-Dec-2001 | 1 | 5×50 |
| 792462 | ** L. nervosum* E. Phillips & Hutch. | C | N | NR | NT | 04-Apr-2014 | 1 | 5×50 |
| 214234 | ** L. nobile* I. Williams | C | W | NR | LC | 13-Dec-2003 | 1 | 5×50 |
| 189255 | *L. procerum* (Salisb. ex Knight) I. Williams | C | W | NR | VU | 18-Mar-2002 | 1 | 5×50 |
| 279394 | ** L. rourkei* I. Williams | C | W | NR | LC | 01-Aug-2005 | 1 | 5×50 |
| 189750 | *L. rubrum* Burm. f. | C | N | NR | LC | 09-Jan-2003 | 1 | 5×50 |
| 189819 | *L. salicifolium* (Salisb.) I. Williams | C | W | NR | LC | 14-Jan-2003 | 1 | 5×50 |
| 190035 | *L. salignum* P.J. Bergius | C | W | R | LC | 07-Feb-2003 | 1 | 5×50 |
| 512228 | *L. sericeum* (Thunb.) R. Br. | S | N | NR | CR | 25-Nov-2008 | 2 | 5×25 |
| 189233 | *L. spissifolium* (Salisb. ex Knight) I. Williams  subsp. *spissifolium* | C | W | R | LC | 18-Mar-2002 | 1 | 5×50 |
| 215091 | *L. stelligerum* I. Williams | C | W | NR | CR | 16-Feb-2004 | 1 | 5×50 |
| 484042 | *L. strobilinum* (L.) Druce | C | W | NR | NT | 22-Jan-2008 | 1 | 5×50 |
| 214050 | *L. teretifolium* (Andrews) I. Williams | C | W | NR | NT | 08-Nov-2003 | 1 | 5×50 |
| 792484 | ** L. thymifolium* (Salisb. ex Knight) I. Williams | S | N | NR | CR | 16-Apr-2014 | 1 | 5×25 |
| 160283 | *L. tinctum* I. Williams | S | N | NR | NT | 26-Oct-2001 | 1 | 5×25 |
| 189222 | ** L. uliginosum* R. Br. subsp. *uliginosum* | C | W | NR | LC | 14-Mar-2002 | 1 | 5×50 |
| 189141 | *L. xanthoconus* (Kuntze) K. Schum. | C | W | NR | LC | 04-Mar-2002 | 1 | 5×50 |

**Table S2.** Model parameters used in the analysis of Experiments 1 and 2. GLM – generalised linear model; GLMM – generalised linear mixed model; Prior FE – fixed effects prior; Prior RE – random effects prior; *N* – normal distribution; *t* – Student’s *t* distribution; *HC* – half-Cauchy distribution; *E* – exponential distribution; Iter – number of iterations per each of five chains; Burn – length of burn in; Thin – thinning rate; PS – total combined number of posterior samples. *N*(0,100) and *HC*(25) are uninformative priors for fixed and random effects, respectively. *N*(0,10) and *E*(1) are weakly informative priors for fixed and random effects, respectively (McElreath 2016). *t*(7,0,2.5) is a weakly informative prior for fixed effects that can be used to obtain finite estimates for logistic regression parameters under complete separation (Ghosh et al*.* 2018).

| **Species** | **Model** | **Prior FE** | **Prior RE** | **Iter (k)** | **Burn (k)** | **Thin (k)** | **PS (k)** |
| --- | --- | --- | --- | --- | --- | --- | --- |
| *L. album* | GLMM | *N*(0,100) | *HC*(25) | 350 | 50 | 100 | 15 |
| *L. argenteum* | GLMM | *N*(0,100) | *HC*(25) | 1550 | 50 | 500 | 15 |
| *L. brunioides* | GLMM | *N*(0,100) | *HC*(25) | 350 | 50 | 100 | 15 |
| *L. chamalaea* | GLMM | *t*(7,0,2.5) | *HC*(25) | 4550 | 50 | 1500 | 15 |
| *L. comosum* | GLM | *t*(7,0,2.5) |  | 500 | 50 | 150 | 15 |
| *L. coniferum* | GLM | *t*(7,0,2.5) |  | 650 | 50 | 200 | 15 |
| *L. corymbosum* | GLMM | *N*(0,100) | *HC*(25) | 110 | 50 | 20 | 15 |
| *L. discolor* | GLMM | *N*(0,10) | *E*(1) | 6050 | 50 | 2000 | 15 |
| *L. dregei* | GLM | *N*(0,100) |  | 500 | 50 | 150 | 15 |
| *L. elimense* | GLM | *N*(0,100) |  | 200 | 50 | 50 | 15 |
| *L. galpinii* | GLMM | *N*(0,100) | *HC*(25) | 650 | 50 | 200 | 15 |
| *L. laxum* | GLMM | *N*(0,100) | *HC*(25) | 350 | 50 | 100 | 15 |
| *L. linifolium* | GLMM | *N*(0,100) | *HC*(25) | 950 | 50 | 300 | 15 |
| *L. loranthifolium* | GLMM | *N*(0,100) | *HC*(25) | 500 | 50 | 150 | 15 |
| *L. microcephalum* | GLM | *t*(7,0,2.5) |  | 650 | 50 | 200 | 15 |
| *L. modestum* | GLMM | *N*(0,100) | *HC*(25) | 2150 | 50 | 700 | 15 |
| *L. muirii* | GLM | *t*(7,0,2.5) |  | 500 | 50 | 150 | 15 |
| *L. nervosum* | GLMM | *N*(0,10) | *HC*(25) | 4550 | 50 | 1500 | 15 |
| *L. procerum* | GLMM | *t*(7,0,2.5) | *E*(1) | 12050 | 50 | 4000 | 15 |
| *L. rubrum* | GLM | *t*(7,0,2.5) |  | 950 | 50 | 300 | 15 |
| *L. salicifolium* | GLM | *t*(7,0,2.5) |  | 950 | 50 | 300 | 15 |
| *L. sericeum* | GLM | *N*(0,100) |  | 80 | 50 | 10 | 15 |
| *L. spissifolium* | GLMM | *t*(7,0,2.5) | *E*(1) | 6050 | 50 | 2000 | 15 |
| *L. stelligerum* | GLM | *t*(7,0,2.5) |  | 1400 | 50 | 450 | 15 |
| *L. thymifolium* | GLMM | *N*(0,100) | *HC*(25) | 200 | 50 | 50 | 15 |
| *L. tinctum* | GLMM | *N*(0,100) | *HC*(25) | 350 | 50 | 100 | 15 |
| *L. uliginosum* | GLMM | *N*(0,100) | *HC*(25) | 3050 | 50 | 1000 | 15 |

**Table S3.** Posterior means, standard deviations (SD) and 95% Bayesian credible intervals of model parameters for Experiment 1. H is the effect of heat, S is the effect of smoke, H×S is their interaction, and *σ* is the standard deviation of the Gaussian random variable used to model the effect of replicate Petri dishes, where applicable (Refer to Table S2 for model parameters). ETI – 95% Equal-Tailed Credible Interval; HDI – 95% Highest-Density Credible Interval (the most credible parameter values); NZE – non-zero effect (the 95% HDI excludes zero): Y – yes, N – no; ESS – effective sample size. Refer to main text for details.

| **Taxon** | **Parameter** | **Mean** | **Mode** | **SD** | **ETI low** | **ETI high** | **HDI low** | **HDI high** | **NZE** | **ESS** |
| --- | --- | --- | --- | --- | --- | --- | --- | --- | --- | --- |
| *L. album* | Intercept | -0.57255 | -0.57650 | 0.20073 | -0.97611 | -0.17736 | -0.96034 | -0.16666 | Y | 15000 |
|  | H | 0.79809 | 0.80419 | 0.28027 | 0.23806 | 1.35598 | 0.22993 | 1.34440 | Y | 15000 |
|  | S | -0.09451 | -0.07450 | 0.28489 | -0.66405 | 0.46990 | -0.65897 | 0.47228 | N | 15000 |
|  | H×S | -0.35057 | -0.33825 | 0.39665 | -1.14460 | 0.44416 | -1.17294 | 0.41288 | N | 15000 |
|  | *σ* | 0.27056 | 0.26183 | 0.13775 | 0.02572 | 0.56114 | 0.00017 | 0.50442 | Y | 14883 |
| *L. argenteum* | Intercept | 2.72109 | 2.63247 | 1.09594 | 0.76963 | 5.14133 | 0.61660 | 4.95148 | Y | 15000 |
|  | H | 0.37649 | 0.61988 | 1.51902 | -2.78162 | 3.32052 | -2.61156 | 3.42580 | N | 15000 |
|  | S | 0.55381 | 0.40489 | 1.51432 | -2.55083 | 3.53078 | -2.55536 | 3.52514 | N | 15000 |
|  | H×S | -0.15718 | -0.27704 | 2.17338 | -4.30770 | 4.35473 | -4.19210 | 4.46019 | N | 15000 |
|  | *σ* | 2.07128 | 1.78262 | 0.65907 | 1.14360 | 3.66619 | 0.99231 | 3.38173 | Y | 15000 |
| *L. brunioides* | Intercept | -3.46030 | -3.37787 | 0.63276 | -4.81471 | -2.29699 | -4.70669 | -2.23508 | Y | 15000 |
|  | H | 1.03874 | 0.95126 | 0.81822 | -0.53084 | 2.70517 | -0.59745 | 2.62341 | N | 15000 |
|  | S | 6.22775 | 6.15928 | 0.85100 | 4.66558 | 8.04102 | 4.61312 | 7.95269 | Y | 15000 |
|  | H×S | -2.35539 | -2.22975 | 1.10672 | -4.61038 | -0.20394 | -4.58873 | -0.18761 | Y | 15000 |
|  | *σ* | 0.94086 | 0.85228 | 0.31505 | 0.44922 | 1.67258 | 0.41012 | 1.59062 | Y | 14203 |
| *L. chamalaea* | Intercept | 1.62181 | 1.55895 | 0.77465 | 0.23587 | 3.34018 | 0.15837 | 3.22497 | Y | 15000 |
|  | H | 0.66125 | 0.82541 | 0.98304 | -1.37549 | 2.56104 | -1.34190 | 2.58048 | N | 15000 |
|  | S | 4.91203 | 4.60046 | 1.59467 | 2.20494 | 8.50665 | 1.83650 | 8.06967 | Y | 15000 |
|  | H×S | -1.46676 | -1.54942 | 1.91835 | -5.48592 | 2.16892 | -5.45939 | 2.17443 | N | 15000 |
|  | *σ* | 1.69455 | 1.41630 | 0.58545 | 0.87307 | 3.11747 | 0.76448 | 2.85376 | Y | 15000 |
| *L. comosum* | Intercept | 7.61309 | 6.35277 | 2.11495 | 4.76099 | 12.78015 | 4.27811 | 11.76006 | Y | 15000 |
|  | H | 0.61649 | 0.40503 | 2.32629 | -3.99754 | 5.30917 | -4.15575 | 5.13141 | N | 15000 |
|  | S | 0.63831 | 0.40586 | 2.30131 | -3.93608 | 5.39194 | -3.70780 | 5.58060 | N | 15000 |
|  | H×S | -2.62587 | -1.99766 | 2.76199 | -8.88246 | 2.17467 | -8.16688 | 2.65608 | N | 14416 |
| *L. coniferum* | Intercept | 6.81027 | 6.12066 | 1.64139 | 4.51928 | 10.86380 | 4.13652 | 10.06524 | Y | 15965 |
|  | H | 2.86443 | 1.91298 | 2.75329 | -1.55723 | 9.34233 | -2.21556 | 8.35831 | N | 16227 |
|  | S | -0.76119 | -0.51908 | 1.90307 | -4.99702 | 2.58919 | -4.63654 | 2.80504 | N | 15000 |
|  | H×S | 1.48250 | 1.01344 | 3.14502 | -4.15291 | 8.33695 | -4.42221 | 7.99102 | N | 15642 |
| *L. corymbosum* | Intercept | -1.18633 | -1.11862 | 0.53831 | -2.26649 | -0.11915 | -2.25972 | -0.11369 | Y | 15000 |
|  | H | 0.43802 | 0.37299 | 0.75542 | -1.07389 | 1.91532 | -1.02628 | 1.95341 | N | 15000 |
|  | S | 2.93245 | 2.96883 | 0.76605 | 1.45906 | 4.47875 | 1.43198 | 4.44463 | Y | 15000 |
|  | H×S | -0.54159 | -0.57690 | 1.06859 | -2.60094 | 1.55560 | -2.61830 | 1.53003 | N | 15000 |
|  | *σ* | 1.05087 | 0.95504 | 0.26875 | 0.63118 | 1.67468 | 0.58170 | 1.58529 | Y | 15000 |
| *L. discolor* | Intercept | 5.22265 | 4.71584 | 1.26771 | 3.16248 | 8.15092 | 2.87435 | 7.69650 | Y | 15000 |
|  | H | 1.57194 | 1.56738 | 1.51959 | -1.34070 | 4.71223 | -1.44053 | 4.59695 | N | 14347 |
|  | S | 1.46793 | 1.38582 | 1.52949 | -1.52992 | 4.59735 | -1.45395 | 4.66388 | N | 15000 |
|  | H×S | -0.65054 | -0.49794 | 2.03270 | -4.73173 | 3.35289 | -4.51155 | 3.55763 | N | 15000 |
|  | *σ* | 2.13755 | 1.74179 | 0.78051 | 1.00617 | 4.02212 | 0.84062 | 3.67234 | Y | 15000 |
| *L. dregei* | Intercept | 3.14903 | 3.13585 | 0.47917 | 2.29240 | 4.18990 | 2.20526 | 4.07451 | Y | 14302 |
|  | H | 0.27049 | 0.22179 | 0.71407 | -1.11969 | 1.72589 | -1.18894 | 1.64918 | N | 15000 |
|  | S | 0.62053 | 0.61786 | 0.78984 | -0.86530 | 2.24539 | -0.88996 | 2.21213 | N | 14474 |
|  | H×S | -1.07578 | -1.04255 | 1.04715 | -3.21838 | 0.93519 | -3.12296 | 1.00219 | N | 15000 |
| *L. elimense* | Intercept | -2.21669 | -2.20372 | 0.22604 | -2.67750 | -1.79618 | -2.67348 | -1.79415 | Y | 15000 |
|  | H | 1.39365 | 1.37285 | 0.26234 | 0.89318 | 1.92230 | 0.88622 | 1.91126 | Y | 18009 |
|  | S | 3.61443 | 3.58017 | 0.28331 | 3.08092 | 4.18119 | 3.07386 | 4.16661 | Y | 15000 |
|  | H×S | 0.08772 | 0.11892 | 0.42313 | -0.73228 | 0.91438 | -0.73117 | 0.91517 | N | 15668 |
| *L. galpinii* | Intercept | 0.39496 | 0.39221 | 0.36293 | -0.31293 | 1.13434 | -0.31695 | 1.12666 | N | 15000 |
|  | H | 0.75076 | 0.83839 | 0.51398 | -0.28568 | 1.76735 | -0.31073 | 1.73233 | N | 15000 |
|  | S | 4.16631 | 4.03838 | 0.78927 | 2.73657 | 5.85069 | 2.63728 | 5.73085 | Y | 15684 |
|  | H×S | -0.73646 | -0.72401 | 1.12318 | -2.94838 | 1.54452 | -2.89205 | 1.57937 | N | 15000 |
|  | *σ* | 0.69552 | 0.60568 | 0.23329 | 0.33798 | 1.24880 | 0.27905 | 1.15071 |  | 15000 |
| *L. laxum* | Intercept | -0.93527 | -0.91387 | 0.37566 | -1.68541 | -0.19725 | -1.68436 | -0.19628 | Y | 15000 |
|  | H | 0.51245 | 0.53267 | 0.52724 | -0.52254 | 1.58070 | -0.48812 | 1.60739 | N | 15000 |
|  | S | 3.38288 | 3.29703 | 0.56968 | 2.30515 | 4.56605 | 2.28529 | 4.53579 | Y | 15000 |
|  | H×S | 0.04251 | -0.00850 | 0.81729 | -1.57226 | 1.66898 | -1.59550 | 1.63180 | N | 15000 |
|  | *σ* | 0.72298 | 0.66258 | 0.23294 | 0.34895 | 1.25883 | 0.31756 | 1.20333 | Y | 15000 |
| *L. linifolium* | Intercept | -1.64019 | -1.62864 | 0.63876 | -2.95219 | -0.39077 | -2.94366 | -0.38520 | Y | 15000 |
|  | H | 0.44622 | 0.42286 | 0.89012 | -1.30699 | 2.22956 | -1.31601 | 2.21529 | N | 15000 |
|  | S | 5.65676 | 5.49367 | 1.01613 | 3.80806 | 7.81516 | 3.63527 | 7.61493 | Y | 15000 |
|  | H×S | -0.55481 | -0.66655 | 1.42326 | -3.31778 | 2.34387 | -3.23775 | 2.39980 | N | 15000 |
|  | *σ* | 1.29446 | 1.10474 | 0.39433 | 0.72421 | 2.24967 | 0.62879 | 2.06844 | Y | 15000 |
| *L. microcephalum* | Intercept | 6.62317 | 5.97251 | 1.47397 | 4.50777 | 10.20359 | 4.22301 | 9.52108 | Y | 13910 |
|  | H | -1.57871 | -1.33112 | 1.58854 | -5.23129 | 1.00351 | -4.71155 | 1.35397 | N | 13753 |
|  | S | 1.67535 | 1.16644 | 2.00907 | -1.86960 | 6.04035 | -2.05459 | 5.78457 | N | 15000 |
|  | H×S | -0.70111 | -0.64309 | 2.15649 | -5.27126 | 3.32126 | -4.87342 | 3.63661 | N | 15000 |
| *L. modestum* | Intercept | 0.60733 | 0.55928 | 0.65308 | -0.68417 | 1.95520 | -0.73212 | 1.89743 | N | 15111 |
|  | H | 1.60863 | 1.48395 | 0.95519 | -0.28045 | 3.60185 | -0.35932 | 3.50646 | N | 15000 |
|  | S | 4.08455 | 3.91894 | 1.25910 | 1.84991 | 6.82395 | 1.78407 | 6.73933 | Y | 15000 |
|  | H×S | -0.53189 | -0.94032 | 2.02929 | -4.34541 | 3.69243 | -4.61598 | 3.38684 | N | 15000 |
|  | *σ* | 1.25225 | 1.09136 | 0.48196 | 0.55241 | 2.42054 | 0.45259 | 2.22691 | Y | 14582 |
| *L. muirii* | Intercept | 5.38216 | 5.12820 | 0.91961 | 3.92787 | 7.48220 | 3.81844 | 7.25405 | Y | 13991 |
|  | H | 0.53226 | 0.35345 | 1.35875 | -2.07045 | 3.36015 | -2.13322 | 3.28996 | N | 14641 |
|  | S | 3.12094 | 2.19754 | 2.29978 | -0.42803 | 8.64629 | -0.79546 | 7.87343 | N | 14519 |
|  | H×S | 1.14243 | 0.84483 | 2.84022 | -4.00002 | 7.32541 | -4.27176 | 6.92432 | N | 15060 |
| *L. nervosum* | Intercept | 4.42861 | 4.19091 | 0.97907 | 2.73381 | 6.57150 | 2.63606 | 6.44061 | Y | 15000 |
|  | H | 0.27355 | 0.27104 | 1.34092 | -2.40703 | 2.93384 | -2.43316 | 2.90199 | N | 15000 |
|  | S | 1.74096 | 1.49526 | 1.62729 | -1.17774 | 5.32546 | -1.52045 | 4.88687 | N | 15000 |
|  | H×S | -3.12421 | -2.85425 | 2.05102 | -7.38920 | 0.77105 | -7.38019 | 0.77252 | N | 15000 |
|  | *σ* | 1.48152 | 1.21619 | 0.68507 | 0.51294 | 3.14130 | 0.33240 | 2.82508 | Y | 15000 |
| *L. procerum* | Intercept | 7.54424 | 6.57385 | 2.06240 | 4.59245 | 12.58288 | 4.13033 | 11.61368 | Y | 14297 |
|  | H | -1.48816 | -1.16712 | 2.22101 | -6.33743 | 2.67733 | -6.06750 | 2.84913 | N | 15000 |
|  | S | 3.58222 | 3.20965 | 2.95604 | -1.24537 | 10.37143 | -1.81874 | 9.56123 | N | 15000 |
|  | H×S | 1.88574 | 0.91358 | 3.42417 | -4.24978 | 9.37754 | -4.70527 | 8.87300 | N | 15000 |
|  | *σ* | 1.75040 | 1.38573 | 0.95356 | 0.20486 | 3.97326 | 0.00106 | 3.48972 | Y | 14042 |
| *L. rubrum* | Intercept | 6.94200 | 6.27676 | 1.68131 | 4.59199 | 10.93714 | 4.29871 | 10.27796 | Y | 15000 |
|  | H | 2.94225 | 2.05953 | 2.78888 | -1.54149 | 9.39079 | -2.14806 | 8.58883 | N | 15000 |
|  | S | -0.71192 | -0.53678 | 1.93398 | -4.98069 | 2.70344 | -4.63887 | 2.89326 | N | 15000 |
|  | H×S | 1.42968 | 0.80321 | 3.17421 | -4.26466 | 8.43519 | -4.59624 | 7.95612 | N | 15000 |
| *L. salicifolium* | Intercept | 6.79997 | 6.03100 | 1.58689 | 4.56117 | 10.62334 | 4.27132 | 9.91943 | Y | 15000 |
|  | H | 3.20236 | 2.06071 | 2.82040 | -1.17922 | 9.72925 | -1.61341 | 8.87874 | N | 15000 |
|  | S | -1.58952 | -1.09585 | 1.73188 | -5.62690 | 1.22830 | -5.12373 | 1.50112 | N | 15000 |
|  | H×S | 1.75357 | 1.35209 | 3.15902 | -3.86254 | 8.55295 | -3.92012 | 8.46512 | N | 15000 |
| *L. spissifolium* | Intercept | 5.02555 | 4.54629 | 1.04658 | 3.47216 | 7.54890 | 3.23709 | 7.11974 | Y | 14567 |
|  | H | -0.00590 | 0.19463 | 1.14926 | -2.37254 | 2.26080 | -2.29290 | 2.31110 | N | 15000 |
|  | S | 2.66831 | 2.60946 | 1.69361 | -0.16065 | 6.57794 | -0.45545 | 6.09937 | N | 15000 |
|  | H×S | -0.82816 | -0.67798 | 1.97786 | -4.88754 | 2.92218 | -4.87223 | 2.92823 | N | 15000 |
|  | *σ* | 1.16534 | 0.90886 | 0.78678 | 0.05178 | 2.98557 | 0.00005 | 2.60184 | Y | 15000 |
| *L. stelligerum* | Intercept | 6.81511 | 6.06154 | 1.58119 | 4.56067 | 10.56061 | 4.31119 | 9.99041 | Y | 16013 |
|  | H | 1.37026 | 1.03743 | 2.00653 | -2.33228 | 5.76829 | -2.62737 | 5.34135 | N | 15000 |
|  | S | -0.97087 | -0.50008 | 1.74451 | -4.89099 | 2.07194 | -4.39291 | 2.34698 | N | 15000 |
|  | H×S | -1.15642 | -0.72600 | 2.21408 | -5.80619 | 3.07898 | -5.77872 | 3.08710 | N | 16411 |
| *L. thymifolium* | Intercept | -1.17126 | -1.10491 | 0.53211 | -2.25683 | -0.14014 | -2.23293 | -0.11939 | Y | 15092 |
|  | H | 2.11400 | 2.02161 | 0.75472 | 0.67135 | 3.71451 | 0.62796 | 3.66279 | Y | 15000 |
|  | S | 4.46110 | 4.20107 | 0.94680 | 2.82896 | 6.53445 | 2.68279 | 6.33143 | Y | 15000 |
|  | H×S | -2.66694 | -2.57472 | 1.26087 | -5.34255 | -0.32458 | -5.11188 | -0.15801 | Y | 15000 |
|  | *σ* | 0.90787 | 0.75543 | 0.39785 | 0.21232 | 1.80479 | 0.16063 | 1.72028 | Y | 14427 |
| *L. tinctum* | Intercept | -0.07638 | -0.06683 | 0.29403 | -0.65198 | 0.50650 | -0.62937 | 0.52200 | N | 15000 |
|  | H | -0.25809 | -0.25473 | 0.41188 | -1.07472 | 0.53789 | -1.07453 | 0.53791 | N | 15000 |
|  | S | -0.25117 | -0.27868 | 0.40733 | -1.05927 | 0.55081 | -1.06489 | 0.54239 | N | 15000 |
|  | H×S | 0.38369 | 0.42222 | 0.57690 | -0.75573 | 1.53678 | -0.73756 | 1.55201 | N | 15000 |
|  | *σ* | 0.36262 | 0.31649 | 0.19756 | 0.02826 | 0.78426 | 0.00033 | 0.70423 | Y | 15000 |
| *L. uliginosum* | Intercept | 2.07379 | 2.02262 | 0.44465 | 1.22406 | 2.99378 | 1.19748 | 2.96189 | Y | 15000 |
|  | H | -0.17028 | -0.21042 | 0.63018 | -1.43223 | 1.11305 | -1.40088 | 1.12992 | N | 15000 |
|  | S | 1.66794 | 1.42760 | 0.76717 | 0.30227 | 3.32955 | 0.23358 | 3.23567 | Y | 15964 |
|  | H×S | 2.30505 | 2.09492 | 1.47105 | -0.35152 | 5.54129 | -0.42227 | 5.40801 | N | 15000 |
|  | *σ* | 0.76501 | 0.67249 | 0.36757 | 0.17157 | 1.63194 | 0.06657 | 1.47177 | Y | 15423 |

**Table S4.** Posterior means, standard deviations (SD) and 95% Bayesian credible intervals of model parameters for Experiment 2. HS is the effect of the combined heat plus smoke treatment and *σ* is the standard deviation of the Gaussian random variable used to model the effect of replicate Petri dishes, where applicable (Refer to Table S2 for model parameters). ETI – 95% Equal-Tailed Credible Interval; HDI – 95% Highest-Density Credible Interval (the most credible parameter values); NZE – non-zero effect (the 95% HDI excludes zero): Y – yes, N – no; ESS – effective sample size. Refer to main text for details.

| **Taxon** | **Parameter** | **Mean** | **Mode** | **SD** | **ETI low** | **ETI high** | **HDI low** | **HDI high** | **NZE** | **ESS** |
| --- | --- | --- | --- | --- | --- | --- | --- | --- | --- | --- |
| *L. loranthifolium* | Intercept | -1.15047 | -1.12825 | 0.44628 | -2.03012 | -0.21145 | -2.08035 | -0.27014 | Y | 14063 |
|  | HS | 2.63467 | 2.63124 | 0.64863 | 1.31130 | 3.95573 | 1.28387 | 3.92437 | Y | 15272 |
|  | *σ* | 0.73739 | 0.64177 | 0.42004 | 0.07068 | 1.73783 | 0.00004 | 1.49420 | Y | 15000 |
| *L. sericeum* | Intercept | -3.31989 | -3.09496 | 0.63514 | -4.72806 | -2.24857 | -4.55259 | -2.12882 | Y | 15000 |
|  | HS | 3.93139 | 3.85535 | 0.68367 | 2.73209 | 5.41221 | 2.66008 | 5.30284 | Y | 15000 |

**Table S5.** Posterior means, standard deviations (SD) and 95% Bayesian credible intervals of germination responses in Experiments 1 and 2. Treatments are control (C), heat (H), smoke (S), and heat plus smoke (HS), and H×S is the effect of the interaction between heat and smoke on the germination probability of the combined heat plus smoke treatment. Refer to main text for details. ETI – 95% Equal-Tailed Credible Interval; HDI – 95% Highest-Density Credible Interval (the 95% most credible parameter values); ESS – effective sample size; PD > ROPE – (control only) proportion of the posterior distribution > ROPE (the Region of Practical Equivalence; the interval [0, 0.5]); Biol. Sig., ‘biological significance’ (control only): NT – ‘biologically non-trivial’ (95% HDI falls completely outside the ROPE), T – ‘biologically trivial’ (95% HDI falls completely inside the ROPE), ? – ‘uncertain’ (95% HDI overlaps the ROPE). Thirteen species (*L. flexuosum, L. foedum, L. eucalyptifolium, L. gandogeri, L. lanigerum, L. laureolum, L. meridianum, L. nobile, L. rourkei, L. salignum, L. strobilinum, L. teretifolium and L. xanthoconus*) that germinated to 100% across all experimental treatments were not subject to statistical analyses and have been omitted.

| **Species** | **Treatment** | **Mean** | **Mode** | **SD** | **ETI low** | **ETI hi** | **HDI low** | **HDI high** | **ESS** | **PD > ROPE ROPE** | | **Biol. Sig** |
| --- | --- | --- | --- | --- | --- | --- | --- | --- | --- | --- | --- | --- |
| *L. album* | C | 0.362 | 0.358 | 0.046 | 0.274 | 0.456 | 0.273 | 0.454 | 15000 | | 0.000 | T |
|  | H | 0.556 | 0.561 | 0.048 | 0.459 | 0.650 | 0.465 | 0.655 | 15000 | |  |  |
|  | S | 0.341 | 0.332 | 0.045 | 0.255 | 0.431 | 0.255 | 0.431 | 13908 | |  |  |
|  | H+S | 0.446 | 0.436 | 0.049 | 0.353 | 0.543 | 0.353 | 0.543 | 15000 | |  |  |
| *L. argenteum* | C | 0.910 | 0.968 | 0.086 | 0.683 | 0.994 | 0.751 | 1.000 | 15000 | | 1.000 | NT |
|  | H | 0.934 | 0.979 | 0.071 | 0.742 | 0.995 | 0.806 | 1.000 | 15000 | |  |  |
|  | S | 0.943 | 0.984 | 0.064 | 0.778 | 0.996 | 0.831 | 1.000 | 15000 | |  |  |
|  | H+S | 0.952 | 0.988 | 0.056 | 0.805 | 0.997 | 0.855 | 1.000 | 15000 | |  |  |
| *L. brunioides* | C | 0.036 | 0.024 | 0.022 | 0.008 | 0.091 | 0.004 | 0.078 | 14363 | | 0.000 | T |
|  | H | 0.090 | 0.068 | 0.045 | 0.029 | 0.199 | 0.020 | 0.175 | 16355 | |  |  |
|  | S | 0.933 | 0.950 | 0.035 | 0.846 | 0.981 | 0.867 | 0.988 | 15000 | |  |  |
|  | H+S | 0.799 | 0.824 | 0.076 | 0.627 | 0.923 | 0.652 | 0.940 | 15000 | |  |  |
| *L. chamalaea* | C | 0.811 | 0.849 | 0.105 | 0.559 | 0.966 | 0.611 | 0.987 | 15000 | | 1.000 | NT |
|  | H | 0.888 | 0.929 | 0.076 | 0.689 | 0.981 | 0.744 | 0.995 | 14450 | |  |  |
|  | S | 0.997 | 1.000 | 0.005 | 0.983 | 1.000 | 0.987 | 1.000 | 15000 | |  |  |
|  | H+S | 0.994 | 0.999 | 0.008 | 0.974 | 1.000 | 0.980 | 1.000 | 15000 | |  |  |
| *L. comosum* | C | 0.998 | 1.000 | 0.002 | 0.992 | 1.000 | 0.994 | 1.000 | 15000 | | 1.000 | NT |
|  | H | 0.999 | 1.000 | 0.002 | 0.992 | 1.000 | 0.995 | 1.000 | 15000 | |  |  |
|  | S | 0.999 | 1.000 | 0.002 | 0.993 | 1.000 | 0.995 | 1.000 | 15000 | |  |  |
|  | H+S | 0.996 | 0.999 | 0.004 | 0.985 | 1.000 | 0.988 | 1.000 | 14573 | |  |  |
| *L. coniferum* | C | 0.997 | 1.000 | 0.003 | 0.989 | 1.000 | 0.992 | 1.000 | 15787 | | 1.000 | NT |
|  | H | 0.999 | 1.000 | 0.001 | 0.995 | 1.000 | 0.997 | 1.000 | 15000 | |  |  |
|  | S | 0.996 | 0.999 | 0.004 | 0.984 | 1.000 | 0.987 | 1.000 | 15364 | |  |  |
|  | H+S | 0.999 | 1.000 | 0.001 | 0.995 | 1.000 | 0.997 | 1.000 | 15000 | |  |  |
| *L. corymbosum* | C | 0.247 | 0.218 | 0.096 | 0.094 | 0.470 | 0.073 | 0.437 | 15000 | | 0.000 | T |
|  | H | 0.331 | 0.332 | 0.112 | 0.135 | 0.578 | 0.119 | 0.551 | 15000 | |  |  |
|  | S | 0.839 | 0.862 | 0.073 | 0.666 | 0.946 | 0.698 | 0.962 | 14311 | |  |  |
|  | H+S | 0.825 | 0.848 | 0.076 | 0.646 | 0.939 | 0.680 | 0.958 | 15000 | |  |  |
| *L. discolor* | C | 0.990 | 0.998 | 0.012 | 0.959 | 1.000 | 0.969 | 1.000 | 15000 | | 1.000 | NT |
|  | H | 0.997 | 1.000 | 0.004 | 0.986 | 1.000 | 0.990 | 1.000 | 15000 | |  |  |
|  | S | 0.997 | 1.000 | 0.005 | 0.985 | 1.000 | 0.989 | 1.000 | 15000 | |  |  |
|  | H+S | 0.998 | 1.000 | 0.003 | 0.990 | 1.000 | 0.993 | 1.000 | 15000 | |  |  |
| *L. dregei* | C | 0.955 | 0.961 | 0.021 | 0.908 | 0.985 | 0.915 | 0.988 | 13876 | | 1.000 | NT |
|  | H | 0.964 | 0.972 | 0.018 | 0.924 | 0.990 | 0.930 | 0.993 | 13375 | |  |  |
|  | S | 0.973 | 0.980 | 0.016 | 0.938 | 0.994 | 0.944 | 0.997 | 15000 | |  |  |
|  | H+S | 0.947 | 0.954 | 0.022 | 0.899 | 0.980 | 0.906 | 0.983 | 15473 | |  |  |
| *L. elimense* | C | 0.100 | 0.098 | 0.021 | 0.064 | 0.142 | 0.062 | 0.140 | 15000 | | 0.000 | T |
|  | H | 0.306 | 0.305 | 0.030 | 0.249 | 0.365 | 0.249 | 0.365 | 15000 | |  |  |
|  | S | 0.801 | 0.807 | 0.026 | 0.748 | 0.848 | 0.749 | 0.849 | 15000 | |  |  |
|  | H+S | 0.945 | 0.948 | 0.016 | 0.912 | 0.970 | 0.915 | 0.972 | 15735 | |  |  |
| *L. galpinii* | C | 0.595 | 0.600 | 0.084 | 0.422 | 0.757 | 0.426 | 0.759 | 15000 | | 0.876 | ? |
|  | H | 0.753 | 0.770 | 0.067 | 0.605 | 0.867 | 0.617 | 0.876 | 15000 | |  |  |
|  | S | 0.987 | 0.992 | 0.009 | 0.965 | 0.998 | 0.970 | 0.999 | 14674 | |  |  |
|  | H+S | 0.987 | 0.992 | 0.009 | 0.965 | 0.998 | 0.970 | 0.999 | 15000 | |  |  |
| *L. laxum* | C | 0.288 | 0.280 | 0.075 | 0.156 | 0.451 | 0.149 | 0.439 | 15000 | | 0.000 | T |
|  | H | 0.399 | 0.400 | 0.085 | 0.235 | 0.580 | 0.228 | 0.570 | 15432 | |  |  |
|  | S | 0.915 | 0.929 | 0.032 | 0.839 | 0.965 | 0.852 | 0.973 | 15947 | |  |  |
|  | H+S | 0.948 | 0.960 | 0.023 | 0.895 | 0.982 | 0.903 | 0.986 | 15000 | |  |  |
| *L. linifolium* | C | 0.180 | 0.126 | 0.091 | 0.050 | 0.404 | 0.030 | 0.357 | 15000 | | 0.000 | T |
|  | H | 0.249 | 0.220 | 0.111 | 0.078 | 0.513 | 0.063 | 0.475 | 15000 | |  |  |
|  | S | 0.977 | 0.988 | 0.019 | 0.930 | 0.997 | 0.944 | 1.000 | 14424 | |  |  |
|  | H+S | 0.974 | 0.986 | 0.021 | 0.922 | 0.997 | 0.936 | 1.000 | 15000 | |  |  |
| *L. loranthifolium* | C | 0.249 | 0.225 | 0.082 | 0.116 | 0.447 | 0.101 | 0.417 | 13945 | | 0.000 | T |
|  | H+S | 0.805 | 0.837 | 0.074 | 0.635 | 0.923 | 0.664 | 0.943 | 14824 | |  |  |
| *L. microcephalum* | C | 0.997 | 1.000 | 0.003 | 0.989 | 1.000 | 0.991 | 1.000 | 13598 | | 1.000 | NT |
|  | H | 0.992 | 0.996 | 0.006 | 0.978 | 0.999 | 0.981 | 1.000 | 15000 | |  |  |
|  | S | 0.999 | 1.000 | 0.002 | 0.994 | 1.000 | 0.996 | 1.000 | 15000 | |  |  |
|  | H+S | 0.996 | 0.999 | 0.004 | 0.985 | 1.000 | 0.988 | 1.000 | 14633 | |  |  |
| *L. modestum* | C | 0.635 | 0.646 | 0.136 | 0.335 | 0.876 | 0.362 | 0.893 | 15154 | | 0.849 | ? |
|  | H | 0.885 | 0.918 | 0.070 | 0.714 | 0.976 | 0.757 | 0.992 | 15000 | |  |  |
|  | S | 0.985 | 0.996 | 0.016 | 0.945 | 0.999 | 0.957 | 1.000 | 15000 | |  |  |
|  | H+S | 0.993 | 0.999 | 0.009 | 0.969 | 1.000 | 0.977 | 1.000 | 15937 | |  |  |
| *L. muirii* | C | 0.994 | 0.997 | 0.005 | 0.981 | 0.999 | 0.983 | 1.000 | 14057 | | 1.000 | NT |
|  | H | 0.996 | 0.999 | 0.004 | 0.984 | 1.000 | 0.987 | 1.000 | 15000 | |  |  |
|  | S | 0.999 | 1.000 | 0.002 | 0.994 | 1.000 | 0.996 | 1.000 | 15665 | |  |  |
|  | H+S | 0.999 | 1.000 | 0.001 | 0.996 | 1.000 | 0.997 | 1.000 | 15354 | |  |  |
| *L. nervosum* | C | 0.982 | 0.994 | 0.019 | 0.939 | 0.999 | 0.953 | 1.000 | 15000 | | 1.000 | NT |
|  | H | 0.986 | 0.995 | 0.016 | 0.948 | 0.999 | 0.961 | 1.000 | 15000 | |  |  |
|  | S | 0.995 | 0.999 | 0.007 | 0.979 | 1.000 | 0.984 | 1.000 | 15000 | |  |  |
|  | H+S | 0.953 | 0.974 | 0.041 | 0.859 | 0.995 | 0.887 | 1.000 | 15000 | |  |  |
| *L. procerum* | C | 0.998 | 1.000 | 0.003 | 0.990 | 1.000 | 0.993 | 1.000 | 15000 | | 1.000 | NT |
|  | H | 0.994 | 0.999 | 0.008 | 0.973 | 1.000 | 0.978 | 1.000 | 15000 | |  |  |
|  | S | 1.000 | 1.000 | 0.001 | 0.998 | 1.000 | 0.999 | 1.000 | 15000 | |  |  |
|  | H+S | 1.000 | 1.000 | 0.001 | 0.997 | 1.000 | 0.998 | 1.000 | 15000 | |  |  |
| *L. rubrum* | C | 0.998 | 1.000 | 0.003 | 0.990 | 1.000 | 0.992 | 1.000 | 15000 | | 1.000 | NT |
|  | H | 0.999 | 1.000 | 0.001 | 0.996 | 1.000 | 0.997 | 1.000 | 15000 | |  |  |
|  | S | 0.997 | 0.999 | 0.004 | 0.987 | 1.000 | 0.989 | 1.000 | 15000 | |  |  |
|  | H+S | 1.000 | 1.000 | 0.001 | 0.996 | 1.000 | 0.997 | 1.000 | 15000 | |  |  |
| *L. salicifolium* | C | 0.998 | 1.000 | 0.003 | 0.990 | 1.000 | 0.992 | 1.000 | 15000 | | 1.000 | NT |
|  | H | 1.000 | 1.000 | 0.001 | 0.996 | 1.000 | 0.998 | 1.000 | 15000 | |  |  |
|  | S | 0.993 | 0.997 | 0.005 | 0.979 | 0.999 | 0.982 | 1.000 | 15000 | |  |  |
|  | H+S | 0.999 | 1.000 | 0.001 | 0.995 | 1.000 | 0.997 | 1.000 | 15000 | |  |  |
| *L. sericeum* | C | 0.041 | 0.031 | 0.023 | 0.009 | 0.095 | 0.005 | 0.086 | 15000 | | 0.000 | T |
|  | H+S | 0.646 | 0.650 | 0.058 | 0.530 | 0.755 | 0.533 | 0.756 | 14298 | |  |  |
| *L. spissifolium* | C | 0.990 | 0.997 | 0.008 | 0.970 | 0.999 | 0.975 | 1.000 | 15000 | | 1.000 | NT |
|  | H | 0.990 | 0.996 | 0.008 | 0.970 | 0.999 | 0.975 | 1.000 | 15000 | |  |  |
|  | S | 0.999 | 1.000 | 0.002 | 0.993 | 1.000 | 0.994 | 1.000 | 15000 | |  |  |
|  | H+S | 0.998 | 1.000 | 0.003 | 0.988 | 1.000 | 0.991 | 1.000 | 15000 | |  |  |
| *L. stelligerum* | C | 0.998 | 1.000 | 0.003 | 0.990 | 1.000 | 0.992 | 1.000 | 15000 | | 1.000 | NT |
|  | H | 0.999 | 1.000 | 0.002 | 0.993 | 1.000 | 0.995 | 1.000 | 15000 | |  |  |
|  | S | 0.995 | 0.999 | 0.004 | 0.984 | 1.000 | 0.987 | 1.000 | 15882 | |  |  |
|  | H+S | 0.996 | 0.999 | 0.004 | 0.984 | 1.000 | 0.987 | 1.000 | 14438 | |  |  |
| *L. thymifolium* | C | 0.249 | 0.239 | 0.095 | 0.095 | 0.465 | 0.079 | 0.439 | 15000 | | 0.000 | T |
|  | H | 0.709 | 0.725 | 0.101 | 0.485 | 0.885 | 0.502 | 0.895 | 15000 | |  |  |
|  | S | 0.955 | 0.975 | 0.031 | 0.878 | 0.993 | 0.897 | 0.999 | 15000 | |  |  |
|  | H+S | 0.928 | 0.952 | 0.043 | 0.822 | 0.984 | 0.848 | 0.993 | 15000 | |  |  |
| *L. tinctum* | C | 0.481 | 0.483 | 0.072 | 0.343 | 0.624 | 0.348 | 0.628 | 15000 | | 0.388 | ? |
|  | H | 0.419 | 0.414 | 0.067 | 0.291 | 0.558 | 0.288 | 0.555 | 15000 | |  |  |
|  | S | 0.420 | 0.413 | 0.067 | 0.293 | 0.555 | 0.289 | 0.549 | 15000 | |  |  |
|  | H+S | 0.451 | 0.448 | 0.070 | 0.315 | 0.593 | 0.307 | 0.584 | 15000 | |  |  |
| *L. uliginosum* | C | 0.881 | 0.888 | 0.047 | 0.773 | 0.952 | 0.792 | 0.962 | 15000 | | 1.000 | NT |
|  | H | 0.862 | 0.870 | 0.053 | 0.742 | 0.945 | 0.763 | 0.957 | 15000 | |  |  |
|  | S | 0.973 | 0.978 | 0.016 | 0.936 | 0.994 | 0.944 | 0.998 | 15000 | |  |  |
|  | H+S | 0.995 | 0.999 | 0.005 | 0.981 | 1.000 | 0.985 | 1.000 | 15000 | |  | T |

**Table S6.** Quantitative and generalised effects of fire-related cues on germination of 40 *Leucadendron* species arranged by germination, seed trait and regeneration syndromes. Germination syndromes: FD (fire cue-dependent) – one or more direct fire cues are required for ≥ 50% germination; FE (fire cue-enhanced) – one or more direct fire cues have a non-trivial (≥ 10%) promotive effect but germination is (> 85%) likely to be ≥ 50% in their absence; FI (fire cue-independent) – direct fire cues have trivial (< 10%) effects on germination and germination is ≥ 50% in their absence; and FU (fire cue effects uncertain) – insufficient evidence to distinguish trivial from non-trivial fire effects, however (with the exception of *L. album* and *L. tinctum* that had low overall germination) direct fire cues are not required for substantial ≥ 50% germination. Seed trait syndromes: SB – seed bank type (C – canopy-stored, S – soil-stored); SM – seed morphology (N – nutlets, W – winged achenes). PS – persistence strategy (NR – non-resprouter, R – resprouter). Quantitative data are modal proportion of viable seeds germinated plus Bayesian 95% Highest-Density Intervals except for 13 species (*) where the probabilities of trivial control germination and trivial treatment effects is inferred to be 0 and 1, respectively, on the basis of 100% germination in all treatments. ‘Effect’ refers to the absolute (*cf* relative) difference in germination between the treatment and control. n/a – individual heat and smoke treatments were not applied. Biological significance (Biol. Sig.): NT – credible evidence of a biologically non-trivial germination response (the 95% HDI excludes the ROPE [0%, 50%] for control germination and [-10%, 10%] for treatment effects); T – credible evidence of a biologically trivial germination response (the 95% HDI is fully contained within the ROPE); ? – insufficient evidence to establish whether control germination and/or treatment effects are biologically trivial or non-trivial. Refer to Materials and Methods for details, and to Table S7 for quantitative data on effect sizes. Life-history and seed trait data obtained from Williams (1972), Rebelo (2001) and Tonnabel et al. (2018).

| **Species** | **Syndrome** | **SB** | **SM** | **PS** | **Control** | **Biol. Sig.** | **Heat effect** | **Biol. Sig.** | **Smoke effect** | **Biol. Sig.** | **Combined effect** | **Biol. Sig.** |
| --- | --- | --- | --- | --- | --- | --- | --- | --- | --- | --- | --- | --- |
| *L. linifolium* | FD | C | N | NR | 0.126 [0.03, 0.357] | **T** | 0.047 [-0.205, 0.371] | **?** | 0.839 [0.617, 0.957] | **NT** | 0.835 [0.611, 0.953] | **NT** |
| *L. elimense* | FD | S | N | NR | 0.098 [0.062, 0.14] | **T** | 0.208 [0.14, 0.278] | **NT** | 0.704 [0.637, 0.766] | **NT** | 0.845 [0.796, 0.892] | **NT** |
| *L. sericeum* | FD | S | N | NR | 0.031 [0.005, 0.086] | **T** | n/a |  | n/a |  | 0.607 [0.481, 0.722] | **NT** |
| *L. corymbosum* | FD | S | N | NR | 0.218 [0.073, 0.437] | **T** | 0.078 [-0.198, 0.385] | **?** | 0.621 [0.349, 0.809] | **NT** | 0.596 [0.33, 0.804] | **NT** |
| *L. laxum* | FD | S | N | NR | 0.28 [0.149, 0.439] | **T** | 0.111 [-0.109, 0.341] | **?** | 0.646 [0.459, 0.779] | **NT** | 0.659 [0.511, 0.817] | **NT** |
| *L. loranthifolium* | FD | S | N | NR | 0.225 [0.101, 0.417] | **T** | n/a |  | n/a |  | 0.573 [0.333, 0.766] | **NT** |
| *L. thymifolium* | FD | S | N | NR | 0.239 [0.079, 0.439] | **T** | 0.466 [0.176, 0.726] | **NT** | 0.723 [0.514, 0.895] | **NT** | 0.693 [0.475, 0.871] | **NT** |
| *L. brunioides* | FD | S | N | R | 0.024 [0.004, 0.078] | **T** | 0.042 [-0.038, 0.152] | **?** | 0.911 [0.816, 0.968] | **NT** | 0.79 [0.607, 0.908] | **NT** |
|  |  |  |  |  |  |  |  |  |  |  |  |  |
| *L. galpinii* | FE | C | N | NR | 0.6 [0.426, 0.759] | **?** | 0.175 [-0.065, 0.363] | **?** | 0.388 [0.226, 0.561] | **NT** | 0.388 [0.224, 0.559] | **NT** |
| *L. modestum* | FE | C | W | NR | 0.646 [0.362, 0.893] | **?** | 0.237 [-0.04, 0.573] | **?** | 0.341 [0.091, 0.624] | **?** | 0.346 [0.107, 0.638] | **NT** |
|  |  |  |  |  |  |  |  |  |  |  |  |  |
| *L. album* | FU | C | N | NR | 0.358 [0.273, 0.454] | **T** | 0.196 [0.064, 0.326] | **?** | -0.017 [-0.151, 0.105] | **?** | 0.081 [-0.046, 0.219] | **?** |
| *L. argenteum* | FU | C | N | NR | 0.968 [0.751, 1] | **NT** | 0.005 [-0.199, 0.256] | **?** | 0.009 [-0.16, 0.266] | **?** | 0.011 [-0.147, 0.264] | **?** |
| *L. nervosum* | FU | C | N | NR | 0.994 [0.953, 1] | **NT** | 0.001 [-0.04, 0.048] | **T** | 0.005 [-0.013, 0.05] | **T** | -0.012 [-0.115, 0.039] | **?** |
| *L. uliginosum* | FU | C | W | NR | 0.888 [0.792, 0.962] | **NT** | -0.024 [-0.169, 0.112] | **?** | 0.076 [0.007, 0.191] | **?** | 0.109 [0.032, 0.202] | **?** |
| *L. chamalaea* | FU | S | N | NR | 0.849 [0.611, 0.987] | **NT** | 0.046 [-0.158, 0.336] | **?** | 0.145 [0.008, 0.382] | **?** | 0.146 [0.009, 0.383] | **?** |
| *L. tinctum* | FU | S | N | NR | 0.483 [0.348, 0.628] | **?** | -0.065 [-0.254, 0.133] | **?** | -0.067 [-0.256, 0.131] | **?** | -0.037 [-0.22, 0.178] | **?** |
|  |  |  |  |  |  |  |  |  |  |  |  |  |
| *L. dregei* | FI | C | N | NR | 0.961 [0.915, 0.988] | **NT** | 0.007 [-0.042, 0.062] | **T** | 0.016 [-0.03, 0.068] | **T** | -0.01 [-0.064, 0.05] | **T** |
| *^*^L. rourkei* | FI | C | W | NR | 1 | **NT** | 0 | **T** | 0 | **T** | 0 | **T** |
| *L. rubrum* | FI | C | N | NR | 1 [0.992, 1] | **NT** | 0 [-0.002, 0.008] | **T** | 0 [-0.011, 0.007] | **T** | 0 [-0.003, 0.009] | **T** |
| *^*^L. lanigerum* | FI | C | W | NR | 1 | **NT** | 0 | **T** | 0 | **T** | 0 | **T** |
| *L. comosum* | FI | C | W | NR | 1 [0.994, 1] | **NT** | 0 [-0.005, 0.007] | **T** | 0 [-0.005, 0.007] | **T** | 0 [-0.013, 0.007] | **T** |
| *L. coniferum* | FI | C | W | NR | 1 [0.992, 1] | **NT** | 0 [-0.002, 0.009] | **T** | 0 [-0.013, 0.008] | **T** | 0 [-0.003, 0.01] | **T** |
| *L. discolor* | FI | C | W | NR | 0.998 [0.969, 1] | **NT** | 0.001 [-0.009, 0.032] | **T** | 0.001 [-0.01, 0.032] | **T** | 0.001 [-0.007, 0.034] | **T** |
| *^*^L. eucalyptifolium* | FI | C | W | NR | 1 | **NT** | 0 | **T** | 0 | **T** | 0 | **T** |
| *^*^L. foedum* | FI | C | W | NR | 1 | **NT** | 0 | **T** | 0 | **T** | 0 | **T** |
| *^*^L. gandogeri* | FI | C | W | NR | 1 | **NT** | 0 | **T** | 0 | **T** | 0 | **T** |
| *^*^L. laureolum* | FI | C | W | NR | 1 | **NT** | 0 | **T** | 0 | **T** | 0 | **T** |
| *^*^L. meridianum* | FI | C | W | NR | 1 | **NT** | 0 | **T** | 0 | **T** | 0 | **T** |
| *L. microcephalum* | FI | C | W | NR | 1 [0.991, 1] | **NT** | -0.003 [-0.019, 0.006] | **T** | 0 [-0.004, 0.009] | **T** | 0 [-0.013, 0.009] | **T** |
| *L. muirii* | FI | C | W | NR | 0.997 [0.983, 1] | **NT** | 0.001 [-0.011, 0.015] | **T** | 0.002 [-0.002, 0.017] | **T** | 0.002 [-0.001, 0.018] | **T** |
| *^*^L. nobile* | FI | C | W | NR | 1 | **NT** | 0 | **T** | 0 | **T** | 0 | **T** |
| *L. procerum* | FI | C | W | NR | 1 [0.993, 1] | **NT** | 0 [-0.023, 0.006] | **T** | 0 [-0.002, 0.008] | **T** | 0 [-0.002, 0.009] | **T** |
| *L. salicifolium* | FI | C | W | NR | 1 [0.992, 1] | **NT** | 0 [-0.002, 0.009] | **T** | -0.002 [-0.018, 0.005] | **T** | 0 [-0.003, 0.009] | **T** |
| *L. stelligerum* | FI | C | W | NR | 1 [0.992, 1] | **NT** | 0 [-0.004, 0.008] | **T** | 0 [-0.013, 0.007] | **T** | 0 [-0.014, 0.008] | **T** |
| *^*^L. strobilinum* | FI | C | W | NR | 1 | **NT** | 0 | **T** | 0 | **T** | 0 | **T** |
| *^*^L. teretifolium* | FI | C | W | NR | 1 | **NT** | 0 | **T** | 0 | **T** | 0 | **T** |
| *^*^L. xanthoconus* | FI | C | W | NR | 1 | **NT** | 0 | **T** | 0 | **T** | 0 | **T** |
| *^*^L. flexuosum* | FI | C | W | R | 1 | **NT** | n/a |  | n/a |  | 0 | **T** |
| *^*^L. salignum* | FI | C | W | R | 1 | **NT** | 0 | **T** | 0 | **T** | 0 | **T** |
| *L. spissifolium* | FI | C | W | R | 0.997 [0.975, 1] | **NT** | 0 [-0.021, 0.021] | **T** | 0.002 [-0.002, 0.025] | **T** | 0.002 [-0.006, 0.026] | **T** |

**Table S7.** Absolute effects of germination treatments in Experiments 1 and 2 expressed as differences in the proportion of viable seeds germinated (summarised in Fig. 1). Treatments are control (C), heat (H), smoke (S), and heat plus smoke (HS), and H×S is the effect of the interaction between heat and smoke on the germination probability of the combined heat plus smoke treatment. Refer to main text for details. ETI – 95% Equal-Tailed Credible Interval; HDI – 95% Highest-Density Credible Interval (the 95% most credible parameter values); ESS – effective sample size; PD < ROPE – proportion of the posterior distribution < ROPE (the Region of Practical Equivalence; the interval [-0.1, 0.1]); PD in ROPE – proportion of the posterior distribution within the ROPE; PD > ROPE – proportion of the posterior distribution > ROPE; HDI < ROPE – proportion of the 95% HDI < ROPE (the Region of Practical Equivalence; the interval [-0.1, 0.1]); HDI in ROPE – proportion of the 95% HDI within the ROPE; HDI > ROPE – proportion of the 95% HDI > ROPE; Biol. Sig., ‘biological significance’ of treatment effects: NT – ‘biologically non-trivial’ (95% HDI falls completely outside the ROPE); T – ‘biologically trivial’ (95% HDI falls completely inside the ROPE); ? – ‘uncertain’ (95% HDI overlaps the ROPE).

| **Species** | **Contrast** | **Mean** | **Mode** | **SD** | **ETI low** | **ETI high** | **HDI low** | **HDI high** | **ESS** | **PD < ROPE** | **PD in ROPE** | **PD > ROPE** | **HDI < ROPE** | **HDI in ROPE** | **HDI > ROPE** | **Biol. Sig.** |
| --- | --- | --- | --- | --- | --- | --- | --- | --- | --- | --- | --- | --- | --- | --- | --- | --- |
| *L. album* | H - C | 0.194 | 0.196 | 0.066 | 0.059 | 0.322 | 0.064 | 0.326 | 15000 | 0.000 | 0.077 | 0.923 | 0.000 | 0.051 | 0.949 | ? |
|  | S - C | -0.021 | -0.017 | 0.064 | -0.150 | 0.107 | -0.151 | 0.105 | 15000 | 0.106 | 0.864 | 0.030 | 0.086 | 0.910 | 0.004 | ? |
|  | HS - C | 0.084 | 0.081 | 0.067 | -0.049 | 0.216 | -0.046 | 0.219 | 15000 | 0.005 | 0.590 | 0.406 | 0.000 | 0.597 | 0.403 | ? |
|  | S - H | -0.215 | -0.213 | 0.065 | -0.343 | -0.084 | -0.345 | -0.086 | 14566 | 0.960 | 0.040 | 0.000 | 0.986 | 0.014 | 0.000 | ? |
|  | HS - H | -0.110 | -0.112 | 0.068 | -0.244 | 0.023 | -0.245 | 0.022 | 15000 | 0.562 | 0.436 | 0.002 | 0.566 | 0.434 | 0.000 | ? |
|  | HS - S | 0.105 | 0.109 | 0.066 | -0.027 | 0.236 | -0.033 | 0.229 | 14635 | 0.003 | 0.460 | 0.537 | 0.000 | 0.466 | 0.534 | ? |
|  | H x S | -0.086 | -0.085 | 0.096 | -0.275 | 0.108 | -0.284 | 0.097 | 15000 | 0.440 | 0.531 | 0.029 | 0.443 | 0.557 | 0.000 | ? |
| *L. argenteum* | H - C | 0.025 | 0.005 | 0.112 | -0.195 | 0.262 | -0.199 | 0.256 | 15000 | 0.077 | 0.752 | 0.171 | 0.056 | 0.792 | 0.152 | ? |
|  | S - C | 0.033 | 0.009 | 0.106 | -0.157 | 0.273 | -0.160 | 0.266 | 15000 | 0.059 | 0.765 | 0.176 | 0.037 | 0.806 | 0.157 | ? |
|  | HS - C | 0.042 | 0.011 | 0.102 | -0.134 | 0.281 | -0.147 | 0.264 | 15000 | 0.040 | 0.768 | 0.192 | 0.020 | 0.809 | 0.172 | ? |
|  | S - H | 0.009 | 0.003 | 0.096 | -0.175 | 0.213 | -0.172 | 0.214 | 15000 | 0.073 | 0.828 | 0.099 | 0.050 | 0.871 | 0.079 | ? |
|  | HS - H | 0.018 | 0.004 | 0.090 | -0.147 | 0.223 | -0.164 | 0.202 | 15000 | 0.050 | 0.842 | 0.108 | 0.032 | 0.887 | 0.082 | ? |
|  | HS - S | 0.009 | 0.001 | 0.085 | -0.156 | 0.191 | -0.143 | 0.200 | 15000 | 0.054 | 0.861 | 0.084 | 0.027 | 0.907 | 0.066 | ? |
|  | H x S | 0.027 | -0.006 | 0.145 | -0.155 | 0.457 | -0.221 | 0.349 | 15000 | 0.061 | 0.794 | 0.145 | 0.053 | 0.836 | 0.111 | ? |
| *L. brunioides* | H - C | 0.054 | 0.042 | 0.050 | -0.027 | 0.168 | -0.038 | 0.152 | 15000 | 0.001 | 0.857 | 0.141 | 0.000 | 0.888 | 0.112 | ? |
|  | S - C | 0.897 | 0.911 | 0.042 | 0.797 | 0.959 | 0.816 | 0.968 | 15000 | 0.000 | 0.000 | 1.000 | 0.000 | 0.000 | 1.000 | NT |
|  | HS - C | 0.763 | 0.790 | 0.079 | 0.587 | 0.896 | 0.607 | 0.908 | 15000 | 0.000 | 0.000 | 1.000 | 0.000 | 0.000 | 1.000 | NT |
|  | S - H | 0.843 | 0.860 | 0.057 | 0.709 | 0.930 | 0.732 | 0.941 | 15000 | 0.000 | 0.000 | 1.000 | 0.000 | 0.000 | 1.000 | NT |
|  | HS - H | 0.709 | 0.729 | 0.088 | 0.511 | 0.862 | 0.534 | 0.878 | 16864 | 0.000 | 0.000 | 1.000 | 0.000 | 0.000 | 1.000 | NT |
|  | HS - S | -0.134 | -0.113 | 0.083 | -0.318 | 0.012 | -0.306 | 0.021 | 15000 | 0.656 | 0.340 | 0.004 | 0.659 | 0.341 | 0.000 | ? |
|  | H x S | -0.168 | -0.148 | 0.084 | -0.348 | -0.019 | -0.343 | -0.016 | 15000 | 0.812 | 0.184 | 0.004 | 0.827 | 0.173 | 0.000 | ? |
| *L. chamalaea* | H - C | 0.077 | 0.046 | 0.121 | -0.158 | 0.336 | -0.158 | 0.336 | 15000 | 0.055 | 0.554 | 0.390 | 0.032 | 0.583 | 0.385 | ? |
|  | S - C | 0.186 | 0.145 | 0.104 | 0.032 | 0.436 | 0.008 | 0.382 | 15000 | 0.000 | 0.213 | 0.787 | 0.000 | 0.223 | 0.777 | ? |
|  | HS - C | 0.183 | 0.146 | 0.104 | 0.029 | 0.437 | 0.009 | 0.383 | 15000 | 0.000 | 0.224 | 0.776 | 0.000 | 0.231 | 0.769 | ? |
|  | S - H | 0.109 | 0.069 | 0.076 | 0.015 | 0.309 | 0.003 | 0.258 | 14463 | 0.000 | 0.551 | 0.449 | 0.000 | 0.576 | 0.424 | ? |
|  | HS - H | 0.106 | 0.063 | 0.076 | 0.014 | 0.305 | 0.002 | 0.254 | 13747 | 0.000 | 0.569 | 0.431 | 0.000 | 0.595 | 0.405 | ? |
|  | HS - S | -0.003 | -0.001 | 0.009 | -0.023 | 0.013 | -0.023 | 0.012 | 15000 | 0.000 | 1.000 | 0.000 | 0.000 | 1.000 | 0.000 | T |
|  | H x S | -0.004 | -0.001 | 0.009 | -0.024 | 0.009 | -0.022 | 0.011 | 15000 | 0.000 | 0.999 | 0.000 | 0.000 | 1.000 | 0.000 | T |
| *L. comosum* | H - C | 0.000 | 0.000 | 0.003 | -0.005 | 0.007 | -0.005 | 0.007 | 15000 | 0.000 | 1.000 | 0.000 | 0.000 | 1.000 | 0.000 | T |
|  | S - C | 0.000 | 0.000 | 0.003 | -0.005 | 0.007 | -0.005 | 0.007 | 14546 | 0.000 | 1.000 | 0.000 | 0.000 | 1.000 | 0.000 | T |
|  | HS - C | -0.002 | 0.000 | 0.005 | -0.014 | 0.006 | -0.013 | 0.007 | 14551 | 0.000 | 1.000 | 0.000 | 0.000 | 1.000 | 0.000 | T |
|  | S - H | 0.000 | 0.000 | 0.003 | -0.006 | 0.007 | -0.007 | 0.007 | 15000 | 0.000 | 1.000 | 0.000 | 0.000 | 1.000 | 0.000 | T |
|  | HS - H | -0.002 | 0.000 | 0.004 | -0.014 | 0.005 | -0.013 | 0.006 | 15000 | 0.000 | 1.000 | 0.000 | 0.000 | 1.000 | 0.000 | T |
|  | HS - S | -0.003 | 0.000 | 0.004 | -0.014 | 0.005 | -0.013 | 0.005 | 15000 | 0.000 | 1.000 | 0.000 | 0.000 | 1.000 | 0.000 | T |
|  | H x S | -0.002 | -0.001 | 0.010 | -0.014 | 0.009 | -0.016 | 0.006 | 15000 | 0.000 | 0.998 | 0.002 | 0.000 | 1.000 | 0.000 | T |
| *L. coniferum* | H - C | 0.002 | 0.000 | 0.003 | -0.002 | 0.010 | -0.002 | 0.009 | 15000 | 0.000 | 1.000 | 0.000 | 0.000 | 1.000 | 0.000 | T |
|  | S - C | -0.002 | 0.000 | 0.005 | -0.014 | 0.007 | -0.013 | 0.008 | 15000 | 0.000 | 1.000 | 0.000 | 0.000 | 1.000 | 0.000 | T |
|  | HS - C | 0.002 | 0.000 | 0.003 | -0.003 | 0.010 | -0.003 | 0.010 | 15000 | 0.000 | 1.000 | 0.000 | 0.000 | 1.000 | 0.000 | T |
|  | S - H | -0.004 | -0.001 | 0.005 | -0.016 | 0.002 | -0.014 | 0.004 | 15000 | 0.000 | 1.000 | 0.000 | 0.000 | 1.000 | 0.000 | T |
|  | HS - H | 0.000 | 0.000 | 0.002 | -0.004 | 0.004 | -0.004 | 0.004 | 15000 | 0.000 | 1.000 | 0.000 | 0.000 | 1.000 | 0.000 | T |
|  | HS - S | 0.004 | 0.001 | 0.004 | -0.002 | 0.016 | -0.002 | 0.015 | 15380 | 0.000 | 1.000 | 0.000 | 0.000 | 1.000 | 0.000 | T |
|  | H x S | 0.002 | 0.000 | 0.012 | -0.003 | 0.013 | -0.005 | 0.009 | 15000 | 0.000 | 0.998 | 0.002 | 0.000 | 1.000 | 0.000 | T |
| *L. corymbosum* | H - C | 0.085 | 0.078 | 0.146 | -0.210 | 0.376 | -0.198 | 0.385 | 15000 | 0.097 | 0.446 | 0.457 | 0.072 | 0.469 | 0.459 | ? |
|  | S - C | 0.592 | 0.621 | 0.120 | 0.327 | 0.793 | 0.349 | 0.809 | 15000 | 0.000 | 0.001 | 0.999 | 0.000 | 0.000 | 1.000 | NT |
|  | HS - C | 0.579 | 0.596 | 0.123 | 0.305 | 0.786 | 0.330 | 0.804 | 15000 | 0.000 | 0.002 | 0.998 | 0.000 | 0.000 | 1.000 | NT |
|  | S - H | 0.507 | 0.536 | 0.134 | 0.219 | 0.747 | 0.244 | 0.762 | 15000 | 0.000 | 0.004 | 0.995 | 0.000 | 0.000 | 1.000 | NT |
|  | HS - H | 0.494 | 0.478 | 0.135 | 0.208 | 0.734 | 0.217 | 0.741 | 15000 | 0.001 | 0.006 | 0.993 | 0.000 | 0.000 | 1.000 | NT |
|  | HS - S | -0.013 | -0.013 | 0.104 | -0.223 | 0.195 | -0.216 | 0.199 | 12671 | 0.182 | 0.700 | 0.118 | 0.162 | 0.737 | 0.101 | ? |
|  | H x S | -0.044 | -0.068 | 0.128 | -0.265 | 0.253 | -0.280 | 0.233 | 15000 | 0.323 | 0.563 | 0.113 | 0.320 | 0.593 | 0.087 | ? |
| *L. discolor* | H - C | 0.007 | 0.001 | 0.012 | -0.005 | 0.037 | -0.009 | 0.032 | 15000 | 0.000 | 0.999 | 0.001 | 0.000 | 1.000 | 0.000 | T |
|  | S - C | 0.007 | 0.001 | 0.012 | -0.007 | 0.037 | -0.010 | 0.032 | 15000 | 0.000 | 0.999 | 0.001 | 0.000 | 1.000 | 0.000 | T |
|  | HS - C | 0.008 | 0.001 | 0.012 | -0.004 | 0.039 | -0.007 | 0.034 | 15000 | 0.000 | 0.999 | 0.001 | 0.000 | 1.000 | 0.000 | T |
|  | S - H | 0.000 | 0.000 | 0.007 | -0.014 | 0.011 | -0.013 | 0.012 | 15000 | 0.000 | 1.000 | 0.000 | 0.000 | 1.000 | 0.000 | T |
|  | HS - H | 0.001 | 0.000 | 0.005 | -0.007 | 0.012 | -0.007 | 0.011 | 15000 | 0.000 | 1.000 | 0.000 | 0.000 | 1.000 | 0.000 | T |
|  | HS - S | 0.001 | 0.000 | 0.006 | -0.007 | 0.014 | -0.008 | 0.012 | 15000 | 0.000 | 1.000 | 0.000 | 0.000 | 1.000 | 0.000 | T |
|  | H x S | 0.000 | 0.000 | 0.006 | -0.008 | 0.006 | -0.008 | 0.006 | 15000 | 0.000 | 1.000 | 0.000 | 0.000 | 1.000 | 0.000 | T |
| *L. dregei* | H - C | 0.009 | 0.007 | 0.026 | -0.041 | 0.063 | -0.042 | 0.062 | 15000 | 0.000 | 0.999 | 0.001 | 0.000 | 1.000 | 0.000 | T |
|  | S - C | 0.019 | 0.016 | 0.025 | -0.028 | 0.070 | -0.030 | 0.068 | 13705 | 0.000 | 0.998 | 0.002 | 0.000 | 1.000 | 0.000 | T |
|  | HS - C | -0.008 | -0.010 | 0.029 | -0.065 | 0.049 | -0.064 | 0.050 | 15000 | 0.002 | 0.998 | 0.000 | 0.000 | 1.000 | 0.000 | T |
|  | S - H | 0.009 | 0.009 | 0.023 | -0.036 | 0.056 | -0.035 | 0.056 | 15000 | 0.000 | 0.999 | 0.001 | 0.000 | 1.000 | 0.000 | T |
|  | HS - H | -0.017 | -0.015 | 0.027 | -0.072 | 0.035 | -0.072 | 0.035 | 15000 | 0.003 | 0.997 | 0.000 | 0.000 | 1.000 | 0.000 | T |
|  | HS - S | -0.026 | -0.023 | 0.025 | -0.080 | 0.022 | -0.077 | 0.025 | 15534 | 0.005 | 0.995 | 0.000 | 0.000 | 1.000 | 0.000 | T |
|  | H x S | -0.027 | -0.030 | 0.033 | -0.084 | 0.050 | -0.089 | 0.040 | 15000 | 0.008 | 0.986 | 0.006 | 0.000 | 1.000 | 0.000 | T |
| *L. elimense* | H - C | 0.206 | 0.208 | 0.035 | 0.138 | 0.275 | 0.140 | 0.278 | 15000 | 0.000 | 0.002 | 0.998 | 0.000 | 0.000 | 1.000 | NT |
|  | S - C | 0.700 | 0.704 | 0.034 | 0.633 | 0.762 | 0.637 | 0.766 | 15000 | 0.000 | 0.000 | 1.000 | 0.000 | 0.000 | 1.000 | NT |
|  | HS - C | 0.845 | 0.845 | 0.027 | 0.793 | 0.890 | 0.796 | 0.892 | 15764 | 0.000 | 0.000 | 1.000 | 0.000 | 0.000 | 1.000 | NT |
|  | S - H | 0.495 | 0.499 | 0.040 | 0.416 | 0.570 | 0.415 | 0.569 | 15000 | 0.000 | 0.000 | 1.000 | 0.000 | 0.000 | 1.000 | NT |
|  | HS - H | 0.639 | 0.645 | 0.034 | 0.572 | 0.702 | 0.573 | 0.702 | 15000 | 0.000 | 0.000 | 1.000 | 0.000 | 0.000 | 1.000 | NT |
|  | HS - S | 0.144 | 0.140 | 0.030 | 0.087 | 0.203 | 0.085 | 0.201 | 16196 | 0.000 | 0.067 | 0.933 | 0.000 | 0.048 | 0.952 | ? |
|  | H x S | 0.005 | 0.007 | 0.023 | -0.039 | 0.051 | -0.040 | 0.050 | 15842 | 0.000 | 1.000 | 0.000 | 0.000 | 1.000 | 0.000 | T |
| *L. galpinii* | H - C | 0.158 | 0.175 | 0.107 | -0.060 | 0.369 | -0.065 | 0.363 | 14634 | 0.013 | 0.261 | 0.726 | 0.000 | 0.265 | 0.735 | ? |
|  | S - C | 0.393 | 0.388 | 0.084 | 0.229 | 0.565 | 0.226 | 0.561 | 15000 | 0.000 | 0.000 | 1.000 | 0.000 | 0.000 | 1.000 | NT |
|  | HS - C | 0.393 | 0.388 | 0.084 | 0.231 | 0.566 | 0.224 | 0.559 | 15000 | 0.000 | 0.001 | 0.999 | 0.000 | 0.000 | 1.000 | NT |
|  | S - H | 0.234 | 0.227 | 0.067 | 0.118 | 0.384 | 0.108 | 0.369 | 15000 | 0.000 | 0.011 | 0.989 | 0.000 | 0.000 | 1.000 | NT |
|  | HS - H | 0.235 | 0.231 | 0.067 | 0.119 | 0.384 | 0.113 | 0.375 | 15000 | 0.000 | 0.011 | 0.989 | 0.000 | 0.000 | 1.000 | NT |
|  | HS - S | 0.000 | 0.000 | 0.012 | -0.025 | 0.025 | -0.024 | 0.026 | 15456 | 0.000 | 1.000 | 0.000 | 0.000 | 1.000 | 0.000 | T |
|  | H x S | -0.006 | -0.003 | 0.011 | -0.030 | 0.016 | -0.031 | 0.014 | 15000 | 0.000 | 1.000 | 0.000 | 0.000 | 1.000 | 0.000 | T |
| *L. laxum* | H - C | 0.111 | 0.111 | 0.113 | -0.114 | 0.336 | -0.109 | 0.341 | 15000 | 0.031 | 0.421 | 0.548 | 0.004 | 0.443 | 0.553 | ? |
|  | S - C | 0.627 | 0.646 | 0.082 | 0.452 | 0.775 | 0.459 | 0.779 | 15000 | 0.000 | 0.000 | 1.000 | 0.000 | 0.000 | 1.000 | NT |
|  | HS - C | 0.660 | 0.659 | 0.078 | 0.490 | 0.803 | 0.511 | 0.817 | 15000 | 0.000 | 0.000 | 1.000 | 0.000 | 0.000 | 1.000 | NT |
|  | S - H | 0.516 | 0.525 | 0.091 | 0.328 | 0.691 | 0.331 | 0.693 | 15561 | 0.000 | 0.000 | 1.000 | 0.000 | 0.000 | 1.000 | NT |
|  | HS - H | 0.549 | 0.550 | 0.088 | 0.367 | 0.717 | 0.373 | 0.723 | 15000 | 0.000 | 0.000 | 1.000 | 0.000 | 0.000 | 1.000 | NT |
|  | HS - S | 0.033 | 0.029 | 0.040 | -0.041 | 0.118 | -0.041 | 0.118 | 15000 | 0.001 | 0.950 | 0.049 | 0.000 | 0.975 | 0.025 | ? |
|  | H x S | 0.007 | -0.004 | 0.046 | -0.068 | 0.115 | -0.076 | 0.099 | 15000 | 0.005 | 0.961 | 0.034 | 0.000 | 1.000 | 0.000 | T |
| *L. linifolium* | H - C | 0.070 | 0.047 | 0.143 | -0.210 | 0.368 | -0.205 | 0.371 | 15000 | 0.096 | 0.513 | 0.391 | 0.073 | 0.540 | 0.387 | ? |
|  | S - C | 0.797 | 0.839 | 0.093 | 0.569 | 0.934 | 0.617 | 0.957 | 15000 | 0.000 | 0.000 | 1.000 | 0.000 | 0.000 | 1.000 | NT |
|  | HS - C | 0.794 | 0.835 | 0.093 | 0.569 | 0.933 | 0.611 | 0.953 | 15000 | 0.000 | 0.000 | 1.000 | 0.000 | 0.000 | 1.000 | NT |
|  | S - H | 0.728 | 0.755 | 0.112 | 0.462 | 0.903 | 0.500 | 0.921 | 15000 | 0.000 | 0.000 | 1.000 | 0.000 | 0.000 | 1.000 | NT |
|  | HS - H | 0.725 | 0.754 | 0.113 | 0.458 | 0.904 | 0.495 | 0.920 | 15000 | 0.000 | 0.000 | 1.000 | 0.000 | 0.000 | 1.000 | NT |
|  | HS - S | -0.003 | -0.001 | 0.028 | -0.059 | 0.051 | -0.055 | 0.054 | 15000 | 0.005 | 0.992 | 0.003 | 0.000 | 1.000 | 0.000 | T |
|  | H x S | -0.005 | -0.005 | 0.038 | -0.065 | 0.078 | -0.075 | 0.062 | 15000 | 0.006 | 0.977 | 0.017 | 0.000 | 1.000 | 0.000 | T |
| *L. loranthifolium* | HS - C | 0.556 | 0.573 | 0.110 | 0.301 | 0.743 | 0.333 | 0.766 | 15282 | 0.000 | 0.003 | 0.997 | 0.000 | 0.000 | 1.000 | NT |
| *L. microcephalum* | H - C | -0.006 | -0.003 | 0.006 | -0.020 | 0.005 | -0.019 | 0.006 | 15000 | 0.000 | 1.000 | 0.000 | 0.000 | 1.000 | 0.000 | T |
|  | S - C | 0.002 | 0.000 | 0.003 | -0.003 | 0.009 | -0.004 | 0.009 | 15000 | 0.000 | 1.000 | 0.000 | 0.000 | 1.000 | 0.000 | T |
|  | HS - C | -0.001 | 0.000 | 0.005 | -0.014 | 0.009 | -0.013 | 0.009 | 14243 | 0.000 | 1.000 | 0.000 | 0.000 | 1.000 | 0.000 | T |
|  | S - H | 0.007 | 0.004 | 0.006 | -0.002 | 0.022 | -0.003 | 0.020 | 15000 | 0.000 | 1.000 | 0.000 | 0.000 | 1.000 | 0.000 | T |
|  | HS - H | 0.004 | 0.002 | 0.007 | -0.009 | 0.019 | -0.009 | 0.019 | 15000 | 0.000 | 1.000 | 0.000 | 0.000 | 1.000 | 0.000 | T |
|  | HS - S | -0.003 | -0.001 | 0.004 | -0.014 | 0.003 | -0.013 | 0.004 | 14529 | 0.000 | 1.000 | 0.000 | 0.000 | 1.000 | 0.000 | T |
|  | H x S | 0.002 | -0.001 | 0.026 | -0.013 | 0.038 | -0.017 | 0.024 | 15000 | 0.000 | 0.993 | 0.007 | 0.000 | 1.000 | 0.000 | T |
| *L. modestum* | H - C | 0.250 | 0.237 | 0.153 | -0.042 | 0.571 | -0.040 | 0.573 | 15628 | 0.011 | 0.136 | 0.853 | 0.000 | 0.128 | 0.872 | ? |
|  | S - C | 0.350 | 0.341 | 0.137 | 0.110 | 0.652 | 0.091 | 0.624 | 15090 | 0.000 | 0.020 | 0.980 | 0.000 | 0.004 | 0.996 | ? |
|  | HS - C | 0.358 | 0.346 | 0.136 | 0.117 | 0.657 | 0.107 | 0.638 | 15142 | 0.000 | 0.016 | 0.984 | 0.000 | 0.000 | 1.000 | NT |
|  | S - H | 0.100 | 0.070 | 0.072 | 0.005 | 0.274 | -0.011 | 0.241 | 15000 | 0.001 | 0.580 | 0.419 | 0.000 | 0.603 | 0.397 | ? |
|  | HS - H | 0.108 | 0.075 | 0.071 | 0.016 | 0.279 | 0.004 | 0.246 | 15000 | 0.000 | 0.534 | 0.466 | 0.000 | 0.554 | 0.446 | ? |
|  | HS - S | 0.008 | 0.002 | 0.019 | -0.021 | 0.050 | -0.024 | 0.046 | 15000 | 0.000 | 0.997 | 0.003 | 0.000 | 1.000 | 0.000 | T |
|  | H x S | -0.002 | -0.001 | 0.015 | -0.027 | 0.019 | -0.027 | 0.019 | 15000 | 0.000 | 0.998 | 0.002 | 0.000 | 1.000 | 0.000 | T |
| *L. muirii* | H - C | 0.002 | 0.001 | 0.006 | -0.010 | 0.016 | -0.011 | 0.015 | 14380 | 0.000 | 1.000 | 0.000 | 0.000 | 1.000 | 0.000 | T |
|  | S - C | 0.005 | 0.002 | 0.005 | -0.001 | 0.018 | -0.002 | 0.017 | 14479 | 0.000 | 1.000 | 0.000 | 0.000 | 1.000 | 0.000 | T |
|  | HS - C | 0.006 | 0.002 | 0.005 | -0.001 | 0.019 | -0.001 | 0.018 | 14261 | 0.000 | 1.000 | 0.000 | 0.000 | 1.000 | 0.000 | T |
|  | S - H | 0.003 | 0.001 | 0.005 | -0.004 | 0.015 | -0.004 | 0.014 | 15000 | 0.000 | 1.000 | 0.000 | 0.000 | 1.000 | 0.000 | T |
|  | HS - H | 0.004 | 0.001 | 0.004 | -0.001 | 0.015 | -0.002 | 0.013 | 14645 | 0.000 | 1.000 | 0.000 | 0.000 | 1.000 | 0.000 | T |
|  | HS - S | 0.000 | 0.000 | 0.002 | -0.003 | 0.005 | -0.003 | 0.005 | 15524 | 0.000 | 1.000 | 0.000 | 0.000 | 1.000 | 0.000 | T |
|  | H x S | 0.000 | 0.000 | 0.003 | -0.003 | 0.005 | -0.004 | 0.005 | 15000 | 0.000 | 1.000 | 0.000 | 0.000 | 1.000 | 0.000 | T |
| *L. nervosum* | H - C | 0.004 | 0.001 | 0.024 | -0.039 | 0.049 | -0.040 | 0.048 | 16508 | 0.003 | 0.992 | 0.005 | 0.000 | 1.000 | 0.000 | T |
|  | S - C | 0.013 | 0.005 | 0.020 | -0.010 | 0.056 | -0.013 | 0.050 | 15000 | 0.000 | 0.994 | 0.006 | 0.000 | 1.000 | 0.000 | T |
|  | HS - C | -0.030 | -0.012 | 0.045 | -0.127 | 0.033 | -0.115 | 0.039 | 15000 | 0.047 | 0.950 | 0.003 | 0.016 | 0.984 | 0.000 | ? |
|  | S - H | 0.009 | 0.003 | 0.017 | -0.013 | 0.048 | -0.017 | 0.041 | 15000 | 0.000 | 0.996 | 0.004 | 0.000 | 1.000 | 0.000 | T |
|  | HS - H | -0.033 | -0.016 | 0.043 | -0.130 | 0.025 | -0.115 | 0.035 | 15000 | 0.051 | 0.947 | 0.002 | 0.017 | 0.983 | 0.000 | ? |
|  | HS - S | -0.043 | -0.021 | 0.041 | -0.138 | 0.001 | -0.115 | 0.009 | 15000 | 0.064 | 0.936 | 0.000 | 0.023 | 0.977 | 0.000 | ? |
|  | H x S | -0.040 | -0.021 | 0.046 | -0.137 | 0.015 | -0.136 | 0.015 | 15000 | 0.063 | 0.932 | 0.005 | 0.040 | 0.960 | 0.000 | ? |
| *L. procerum* | H - C | -0.004 | 0.000 | 0.008 | -0.025 | 0.005 | -0.023 | 0.006 | 15000 | 0.000 | 1.000 | 0.000 | 0.000 | 1.000 | 0.000 | T |
|  | S - C | 0.002 | 0.000 | 0.003 | -0.001 | 0.009 | -0.002 | 0.008 | 15000 | 0.000 | 1.000 | 0.000 | 0.000 | 1.000 | 0.000 | T |
|  | HS - C | 0.001 | 0.000 | 0.003 | -0.002 | 0.010 | -0.002 | 0.009 | 15000 | 0.000 | 1.000 | 0.000 | 0.000 | 1.000 | 0.000 | T |
|  | S - H | 0.006 | 0.001 | 0.008 | 0.000 | 0.027 | -0.002 | 0.022 | 15000 | 0.000 | 1.000 | 0.000 | 0.000 | 1.000 | 0.000 | T |
|  | HS - H | 0.006 | 0.000 | 0.008 | 0.000 | 0.026 | -0.001 | 0.023 | 15000 | 0.000 | 1.000 | 0.000 | 0.000 | 1.000 | 0.000 | T |
|  | HS - S | 0.000 | 0.000 | 0.001 | -0.002 | 0.002 | -0.002 | 0.002 | 15856 | 0.000 | 1.000 | 0.000 | 0.000 | 1.000 | 0.000 | T |
|  | H x S | 0.002 | 0.001 | 0.021 | -0.002 | 0.015 | -0.004 | 0.009 | 15000 | 0.000 | 0.996 | 0.004 | 0.000 | 1.000 | 0.000 | T |
| *L. rubrum* | H - C | 0.002 | 0.000 | 0.003 | -0.002 | 0.009 | -0.002 | 0.008 | 15000 | 0.000 | 1.000 | 0.000 | 0.000 | 1.000 | 0.000 | T |
|  | S - C | -0.001 | 0.000 | 0.004 | -0.011 | 0.007 | -0.011 | 0.007 | 15754 | 0.000 | 1.000 | 0.000 | 0.000 | 1.000 | 0.000 | T |
|  | HS - C | 0.002 | 0.000 | 0.003 | -0.003 | 0.010 | -0.003 | 0.009 | 15000 | 0.000 | 1.000 | 0.000 | 0.000 | 1.000 | 0.000 | T |
|  | S - H | -0.003 | -0.001 | 0.004 | -0.013 | 0.002 | -0.012 | 0.002 | 15000 | 0.000 | 1.000 | 0.000 | 0.000 | 1.000 | 0.000 | T |
|  | HS - H | 0.000 | 0.000 | 0.002 | -0.003 | 0.003 | -0.003 | 0.003 | 15000 | 0.000 | 1.000 | 0.000 | 0.000 | 1.000 | 0.000 | T |
|  | HS - S | 0.003 | 0.001 | 0.004 | -0.001 | 0.013 | -0.002 | 0.012 | 15000 | 0.000 | 1.000 | 0.000 | 0.000 | 1.000 | 0.000 | T |
|  | H x S | 0.002 | 0.000 | 0.016 | -0.003 | 0.011 | -0.004 | 0.007 | 15000 | 0.000 | 0.997 | 0.003 | 0.000 | 1.000 | 0.000 | T |
| *L. salicifolium* | H - C | 0.002 | 0.000 | 0.003 | -0.001 | 0.009 | -0.002 | 0.009 | 15000 | 0.000 | 1.000 | 0.000 | 0.000 | 1.000 | 0.000 | T |
|  | S - C | -0.005 | -0.002 | 0.006 | -0.018 | 0.005 | -0.018 | 0.005 | 15000 | 0.000 | 1.000 | 0.000 | 0.000 | 1.000 | 0.000 | T |
|  | HS - C | 0.002 | 0.000 | 0.003 | -0.003 | 0.010 | -0.003 | 0.009 | 15000 | 0.000 | 1.000 | 0.000 | 0.000 | 1.000 | 0.000 | T |
|  | S - H | -0.007 | -0.003 | 0.005 | -0.020 | 0.000 | -0.019 | 0.001 | 15000 | 0.000 | 1.000 | 0.000 | 0.000 | 1.000 | 0.000 | T |
|  | HS - H | 0.000 | 0.000 | 0.002 | -0.004 | 0.003 | -0.004 | 0.003 | 14560 | 0.000 | 1.000 | 0.000 | 0.000 | 1.000 | 0.000 | T |
|  | HS - S | 0.007 | 0.003 | 0.005 | 0.000 | 0.020 | -0.001 | 0.019 | 15000 | 0.000 | 1.000 | 0.000 | 0.000 | 1.000 | 0.000 | T |
|  | H x S | 0.003 | 0.000 | 0.023 | -0.003 | 0.018 | -0.005 | 0.012 | 15000 | 0.000 | 0.996 | 0.004 | 0.000 | 1.000 | 0.000 | T |
| *L. sericeum* | HS - C | 0.605 | 0.607 | 0.062 | 0.478 | 0.720 | 0.481 | 0.722 | 15000 | 0.000 | 0.000 | 1.000 | 0.000 | 0.000 | 1.000 | NT |
| *L. spissifolium* | H - C | 0.000 | 0.000 | 0.011 | -0.022 | 0.021 | -0.021 | 0.021 | 15000 | 0.000 | 1.000 | 0.000 | 0.000 | 1.000 | 0.000 | T |
|  | S - C | 0.008 | 0.002 | 0.008 | 0.000 | 0.028 | -0.002 | 0.025 | 15000 | 0.000 | 1.000 | 0.000 | 0.000 | 1.000 | 0.000 | T |
|  | HS - C | 0.007 | 0.002 | 0.009 | -0.005 | 0.028 | -0.006 | 0.026 | 15000 | 0.000 | 1.000 | 0.000 | 0.000 | 1.000 | 0.000 | T |
|  | S - H | 0.008 | 0.003 | 0.009 | -0.002 | 0.029 | -0.003 | 0.026 | 15000 | 0.000 | 1.000 | 0.000 | 0.000 | 1.000 | 0.000 | T |
|  | HS - H | 0.007 | 0.002 | 0.009 | -0.004 | 0.027 | -0.006 | 0.026 | 15000 | 0.000 | 1.000 | 0.000 | 0.000 | 1.000 | 0.000 | T |
|  | HS - S | -0.001 | 0.000 | 0.004 | -0.010 | 0.005 | -0.009 | 0.006 | 15000 | 0.000 | 1.000 | 0.000 | 0.000 | 1.000 | 0.000 | T |
|  | H x S | -0.001 | 0.000 | 0.005 | -0.010 | 0.007 | -0.010 | 0.007 | 15414 | 0.000 | 1.000 | 0.000 | 0.000 | 1.000 | 0.000 | T |
| *L. stelligerum* | H - C | 0.001 | 0.000 | 0.003 | -0.004 | 0.009 | -0.004 | 0.008 | 15000 | 0.000 | 1.000 | 0.000 | 0.000 | 1.000 | 0.000 | T |
|  | S - C | -0.002 | 0.000 | 0.005 | -0.013 | 0.006 | -0.013 | 0.007 | 15411 | 0.000 | 1.000 | 0.000 | 0.000 | 1.000 | 0.000 | T |
|  | HS - C | -0.002 | 0.000 | 0.005 | -0.014 | 0.008 | -0.014 | 0.008 | 15409 | 0.000 | 1.000 | 0.000 | 0.000 | 1.000 | 0.000 | T |
|  | S - H | -0.003 | -0.001 | 0.005 | -0.015 | 0.004 | -0.015 | 0.004 | 15000 | 0.000 | 1.000 | 0.000 | 0.000 | 1.000 | 0.000 | T |
|  | HS - H | -0.003 | 0.000 | 0.004 | -0.015 | 0.004 | -0.013 | 0.005 | 15027 | 0.000 | 1.000 | 0.000 | 0.000 | 1.000 | 0.000 | T |
|  | HS - S | 0.000 | 0.000 | 0.006 | -0.012 | 0.012 | -0.012 | 0.012 | 15000 | 0.000 | 1.000 | 0.000 | 0.000 | 1.000 | 0.000 | T |
|  | H x S | 0.001 | -0.001 | 0.026 | -0.013 | 0.028 | -0.018 | 0.017 | 15000 | 0.000 | 0.994 | 0.006 | 0.000 | 1.000 | 0.000 | T |
| *L. thymifolium* | H - C | 0.460 | 0.466 | 0.140 | 0.157 | 0.715 | 0.176 | 0.726 | 15000 | 0.002 | 0.012 | 0.986 | 0.000 | 0.000 | 1.000 | NT |
|  | S - C | 0.706 | 0.723 | 0.099 | 0.487 | 0.876 | 0.514 | 0.895 | 15000 | 0.000 | 0.000 | 1.000 | 0.000 | 0.000 | 1.000 | NT |
|  | HS - C | 0.680 | 0.693 | 0.104 | 0.445 | 0.856 | 0.475 | 0.871 | 15000 | 0.000 | 0.000 | 1.000 | 0.000 | 0.000 | 1.000 | NT |
|  | S - H | 0.246 | 0.231 | 0.105 | 0.064 | 0.479 | 0.050 | 0.461 | 15000 | 0.001 | 0.062 | 0.937 | 0.000 | 0.048 | 0.952 | ? |
|  | HS - H | 0.220 | 0.215 | 0.110 | 0.021 | 0.456 | 0.009 | 0.442 | 15000 | 0.002 | 0.121 | 0.877 | 0.000 | 0.110 | 0.890 | ? |
|  | HS - S | -0.026 | -0.012 | 0.053 | -0.145 | 0.068 | -0.139 | 0.071 | 15000 | 0.073 | 0.918 | 0.009 | 0.047 | 0.953 | 0.000 | ? |
|  | H x S | -0.064 | -0.041 | 0.045 | -0.172 | -0.006 | -0.152 | 0.003 | 15000 | 0.164 | 0.835 | 0.001 | 0.131 | 0.869 | 0.000 | ? |
| *L. tinctum* | H - C | -0.063 | -0.065 | 0.099 | -0.259 | 0.131 | -0.254 | 0.133 | 15000 | 0.348 | 0.602 | 0.050 | 0.339 | 0.634 | 0.028 | ? |
|  | S - C | -0.061 | -0.067 | 0.098 | -0.254 | 0.134 | -0.256 | 0.131 | 15000 | 0.344 | 0.605 | 0.050 | 0.338 | 0.637 | 0.025 | ? |
|  | HS - C | -0.031 | -0.037 | 0.100 | -0.229 | 0.169 | -0.220 | 0.178 | 15000 | 0.234 | 0.677 | 0.089 | 0.215 | 0.712 | 0.072 | ? |
|  | S - H | 0.002 | 0.007 | 0.095 | -0.184 | 0.188 | -0.182 | 0.189 | 15000 | 0.136 | 0.721 | 0.143 | 0.115 | 0.759 | 0.126 | ? |
|  | HS - H | 0.032 | 0.029 | 0.097 | -0.160 | 0.225 | -0.157 | 0.226 | 15000 | 0.084 | 0.682 | 0.235 | 0.060 | 0.717 | 0.222 | ? |
|  | HS - S | 0.030 | 0.025 | 0.097 | -0.161 | 0.223 | -0.163 | 0.219 | 15000 | 0.086 | 0.685 | 0.229 | 0.066 | 0.721 | 0.213 | ? |
|  | H x S | 0.086 | 0.105 | 0.130 | -0.182 | 0.332 | -0.182 | 0.332 | 15000 | 0.080 | 0.451 | 0.469 | 0.058 | 0.475 | 0.467 | ? |
| *L. uliginosum* | H - C | -0.019 | -0.024 | 0.071 | -0.162 | 0.122 | -0.169 | 0.112 | 15000 | 0.097 | 0.864 | 0.039 | 0.080 | 0.909 | 0.010 | ? |
|  | S - C | 0.092 | 0.076 | 0.050 | 0.015 | 0.207 | 0.007 | 0.191 | 15000 | 0.000 | 0.631 | 0.369 | 0.000 | 0.648 | 0.352 | ? |
|  | HS - C | 0.114 | 0.109 | 0.047 | 0.042 | 0.223 | 0.032 | 0.202 | 15000 | 0.000 | 0.412 | 0.588 | 0.000 | 0.424 | 0.576 | ? |
|  | S - H | 0.111 | 0.104 | 0.055 | 0.024 | 0.235 | 0.011 | 0.217 | 15000 | 0.000 | 0.461 | 0.539 | 0.000 | 0.473 | 0.527 | ? |
|  | HS - H | 0.133 | 0.118 | 0.053 | 0.050 | 0.254 | 0.041 | 0.236 | 15000 | 0.000 | 0.266 | 0.734 | 0.000 | 0.265 | 0.735 | ? |
|  | HS - S | 0.022 | 0.019 | 0.016 | -0.002 | 0.060 | -0.005 | 0.055 | 15000 | 0.000 | 0.998 | 0.002 | 0.000 | 1.000 | 0.000 | T |
|  | H x S | 0.033 | 0.013 | 0.035 | -0.002 | 0.117 | -0.010 | 0.095 | 15000 | 0.000 | 0.961 | 0.039 | 0.000 | 1.000 | 0.000 | T |

**Fig S1** Illustration of the HDI + ROPE decision rule used to distinguish biologically trivial from non-trivial treatment effects. Data presented are modal % change in germination (circles) with Bayesian 95% Highest-Density Intervals (HDI) (the 95% most credible/highest probability density parameter values) from pairwise treatment contrasts (*n* = 177, Table S7). Dashed vertical line indicates zero effect. Hollow circles indicate HDIs that exclude zero (analogous but not equivalent to ‘statistically significant’ in frequentist NHST) while filled circles indicate HDIs that include zero (analogous but not equivalent to ‘statistically non-significant’ in frequentist NHST). Light grey shading indicates the Region of Practical Equivalence (ROPE) – effect sizes regarded as only trivially different from zero in the present study. Effects with HDIs completely contained within the ROPE were classified as ‘biologically trivial’; those falling outside the ROPE as ‘biologically non-trivial’; and those overlapping the ROPE as ‘uncertain’. Refer to Materials and Methods for further details

**References**

Baskin CC, Baskin JM (2003) When breaking seed dormancy is a problem try a move-along experiment. Native Plants J 4:17-21 doi: 10.3368/npj.4.1.17

Baskin CC, Baskin JM (2014) Seeds: ecology, biogeography and evolution of dormancy and germination, 2nd edn. Academic Press, San Diego

Ghosh J, Li Y, Mitra R (2018) On the use of Cauchy prior distributions for Bayesian logistic regression. Bayesian Anal 13:359-383 doi: 10.1214/17-BA1051

McElreath R (2016) Statistical rethinking: a Bayesian course with examples in R and Stan. Chapman & Hall/CRC texts in statistical science series, Boca Raton

Rebelo T (2001) Proteas: a field guide to the proteas of Southern Africa, 2nd edn. Fernwood Press, Cape Town

SANBI (2017) Red List of South African Plants version 2017.1. http://redlist.sanbi.org/ (accessed 18 November 2019)

Tonnabel J, Schurr FM, Boucher F, Thuiller W, Renaud J, Douzery EJP, Ronce O (2018) Life-history traits evolved jointly with climatic niche and disturbance regime in the genus *Leucadendron* (Proteaceae). Am Nat 191:220-234 doi: 10.1086/695283

Williams IJM (1972) A revision of the genus *Leucadendron* (Proteaceae). Contrib Bolus Herb 3:1–425
