# Supplementary figures and images for: Fire-mediated germination syndromes in Leucadendron (Proteaceae) and their functional correlates
Source: Oecologia. 2021 Jun 23;196(2):589–604. doi: 10.1007/s00442-021-04947-2 (PMC8241639; doi:10.1007/s00442-021-04947-2)

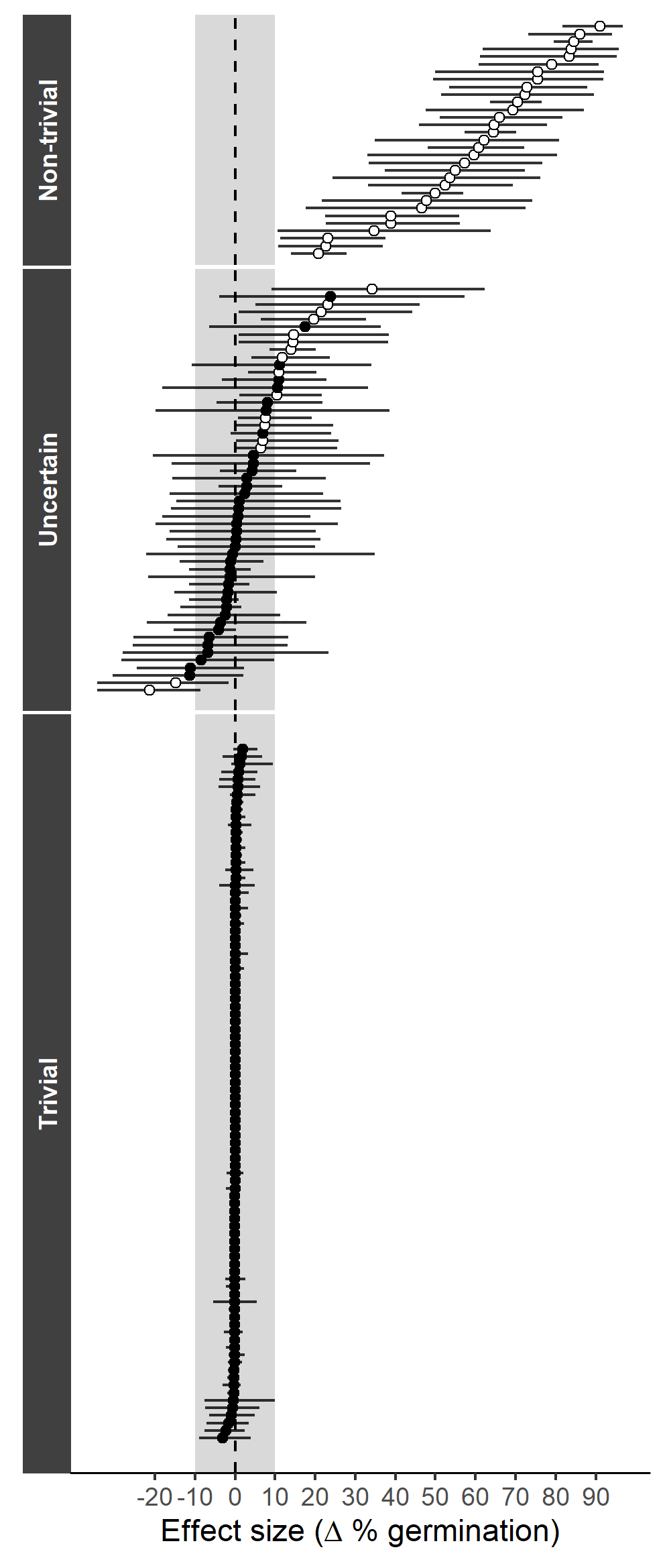

Supplement: Supplementary file 2 — Supplementary file2 (TIF 53 KB) [file 442_2021_4947_MOESM2_ESM.tif]
